# Unlocking the future of precision manufacturing: A comprehensive exploration of 3D printing with fiber-reinforced composites in aerospace, automotive, medical, and consumer industries

**DOI:** 10.1016/j.heliyon.2024.e27328

**Published:** 2024-03-05

**Authors:** Noshin Tasnim Tuli, Sinthea Khatun, Adib Bin Rashid

**Affiliations:** Industrial and Production Engineering Department, Military Institute of Science and Technology (MIST), Dhaka-1216, Bangladesh

**Keywords:** 3D printing, Additive manufacturing, Fiber reinforced composites, Fused deposition modeling, Polymer matrix

## Abstract

Rapid advancements in the field of 3D printing in the last several decades have made it possible to produce complex and unique parts with remarkable precision and accuracy. Investigating the use of 3D printing to create various high-performance materials is a relatively new field that is expanding exponentially worldwide. Automobile, biomedical, construction, aerospace, electronics, and metal and alloy industries are among the most prolific users of 3D printing technology. Modern 3D printing technologies, such as polymer matrices that use fiber-reinforced composites (FRCs) to enhance the mechanical qualities of printed components greatly, have been useful to several industries. High stiffness and tensile strength lightweight components are developed from these materials. Fiber-reinforced composites have a wide range of applications, such as military vehicles, fighter aircraft, underwater structures, shelters, and warfare equipment. Fabricating FRCs using fused deposition modeling (FDM) is also advantageous over other 3D printing methods due to its low cost and ease of operation. The impact of different continuous fiber and matrix polymer selections on FRC performance is covered in this review paper. We will also evaluate the important parameters influencing FRC characteristics and review the most recent equipment and methods for fabricating FRCs. Furthermore, the challenges associated with 3D printing fiber-reinforced composites are covered. The constraints of present technology have also been used to identify future research areas.

## 1 Introduction

3D printing is an approach for fabricating objects through depositing material in specific, ordered layers from a CAD model and is widely recognized as a potent method in the modern manufacturing sector [1]. Increased accessibility and a wider range of applications have resulted from numerous recent developments in additive manufacturing technology. Additive fabrication, Additive manufacturing, additive processes, additive layer fabrication, layered manufacturing, additive techniques, fast prototyping, solid freeform fabrication (SFF), and rapid manufacturing are some of the other names for 3D printing [2]. A wide variety of sectors, from agriculture and medicine to transportation and aerospace, are increasingly turning to 3D printing technology for mass customization and manufacture of open-source models [3]. It allows for extensive design freedom and the possibility of fabricating complicated item shapes from a combination of materials [4].

The term “three-dimensional printing” (3DP) is currently an area of interest for scientists fascinated with materials. A revolution in the production industries' ability to design next-generation high-performance materials is expected to result from this technology, which has seen tremendous growth in recent years. An example of how the market has surpassed expectations is additive manufacturing (AM). 3D printing is altering the manufacturing industry in revolutionary ways. It renders low-volume production cheaper by doing away with costly machinery and molds. It additionally allows for widespread customization by making each product entirely adaptable. It creates a “virtual stock” by replacing mechanical parts with electronic files that can be created on demand [5].

Additive manufacturing (AM) is a versatile method of production, and fiber-reinforced additive manufacturing (FRAM) takes those advantages one step further by incorporating the strengths of composite materials. The human labor involved in traditional composite manufacturing, such as mold preparation, is eliminated, allowing for the simultaneous fabrication of critically shaped parts. AM makes rapid production of unique components with minimum human involvement feasible [6].

Fibers can be placed precisely and in a variety of orientations and patterns using additive manufacturing, leading to composites with enhanced mechanical qualities and endurance. The use of continuous fiber reinforcing is a significant development in additive manufacturing for FRP composites. In the continuous fiber reinforcement method, the matrix of polymers is fortified with the help of long, continuous fibers. This strategy enhances composites' strength, stiffness, impact resistance, and fatigue life. Hybrid additive manufacturing procedures, which utilize multiple approaches to create advanced structures, are another recent breakthrough. Large-scale FRP composite structures with high precision and fiber alignment have been made using 3D printing and automated fiber placement (AFP). The use of this method permits the construction of intricate structures that are both sturdy and lightweight [[7], [8]].

Moreover, fabricating fiber composites from a combination of two different materials is a novel application of additive manufacturing. Traditional composite manufacturing frequently employed additive methods such as manual layup, resin transfer molding, fiber substitution, and automatic filament winding. A fiber-replacement & automatic filament-winding method is used to boost both the component integrity & manufacturing speed. Due to limited printable material and printer compatibility, 3D printing originated with single-material printing. Single-material printing reduces the strength of additive-created products, whereas printing improves composites' mechanical characteristics. Layered composite material fabrication was also used to create multi-material composites with numerous printing heads. Multilateral printing enhanced mechanical qualities and expanded AM processes [[9], [10]].

Different reinforcements are used to enhance the performance of the plastic composite; for instance, carbon black, chopped fibers, platelets, and polymer fibrils are mixed with a thermoplastic matrix and then extruded together during printing. The performance of these composites significantly depends on the fiber orientation in the plastic and fiber volume fraction (FVF). However, they still show inferior mechanical performance compared to traditional fiber-reinforced composites. Therefore, continuous fiber-reinforced composites are a pressing need to widen the application of 3D-printed FMD technology for designing high-performance composites. The technology available in the market with this feature is known as continuous filament fabrication (CFF).

**Fig. 1:**
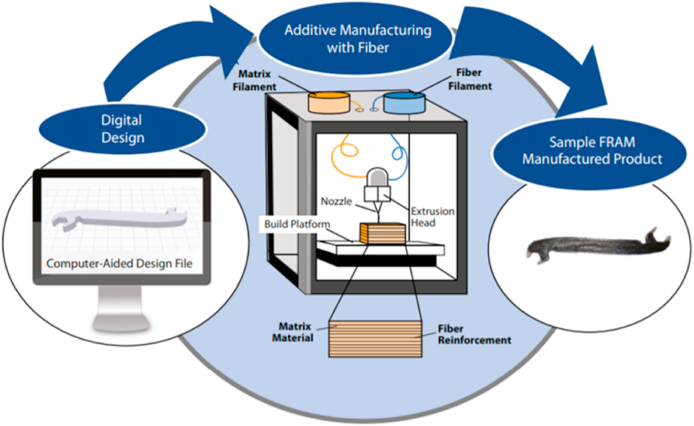
Graphical representation layout of Fiber reinforced additive manufacturing (FRAM) [13].

The foundation of FRPs is a softer polymer matrix with extremely strain-resistant fibers inserted in it. Fiberglass (FG), carbon fibers, textiles, Kevlar, and basalt are the most common types of reinforcement employed. All sorts of natural fibers, from paper and wood to asbestos, can be used using this technique. Polyester, Nylon, epoxy, vinyl ester, aluminum oxide, titanium, and aluminum alloys constitute all matrix-based substances employed to make CMs. The mechanical and physical characteristics of composite materials are significantly influenced by fiber volume, length, type, and orientation [6]. Soft and rigid body systems create structural supports, heat exchange, electrical micromachines, optical devices, surface alterations, and biological applications [2]. Pressure resistance is improved due to the fibers, and the polymer matrix assists in protecting them as well as distributing the load [11]. Hence, it is versatile in design, has higher integrity and damage tolerance, a tremendous boost in strength, from 140% to 335%, and is less expensive. The current industrial situation shows FRP composite material being used with AM technology to produce a manufactured product. Manufacturing FRP parts employs the SLA method, while most businesses rely on the Fused Layer Modeling method (FLM). The mechanical capabilities of FRP composite components are enhanced when the fibers are more extensive and continuous [12]. Fig. 1 displays a simplified representation of the FRAM method.

There are two primary fiber-reinforced composites: natural and synthetic [14]. Fiber-reinforced polymer composites historically used synthetic fibers. Synthetic fibers are lab-made. These fibers form a continuous filament after extrusion. Artificial fibers are used in mechanical, thermal, and chemical applications. Man-made fibers include carbon, glass, quartz, ceramic, aramid, and nylon [15]. The diverse benefits include fracture toughness, high strength, durability, impact resistance, and stiffness. However, synthetic fibers are made from nonrenewable and petroleum-based raw ingredients, none breaking down in the environment. Current environmental regulation has a problem with artificial fibers since they are difficult to recycle, biodegrade, and reuse [16].

On the other hand, natural fibers have several benefits because they are made from renewable resources like plants and animals and are therefore biodegradable and biocompatible [17]. Natural fiber-reinforced composites (NFRCs) are being used by companies and educational institutions for a variety of purposes to lessen their environmental effect and satisfy the increasing need for textiles [18]. Due to the reduced density of natural fibers compared to synthetic fibers, NFRCs can also be fabricated (glass fiber: 2.4 g/cm^3^). Natural fibers plus thermosetting or thermoplastic polymers make NFRCs “green composites,” “eco composites,” and “biocomposites.” They consist of a polymer matrix (biopolymer or petroleum) & a reinforcing element (particles or fibers). NFRCs are categorized as green, semi-green, or hybrid by the number of natural materials employed as reinforcement medium. Green composites, such as polylactic acid (PLA) and hemp, are made from renewable polymer matrices and fibers. Natural fibers have undesired qualities that negatively affect the mechanical properties of the composite, making it challenging to create a composite material composed solely of biodegradable components. Composites made from a combination of natural fibers and a polymeric matrix are the subject of intense research and development.

Furthermore, weak interlaminar bonding performance is a major issue that prevents 3D-printed continuous FRPCs from being used effectively. As-deposited layer imperfections, such as pores or voids, play a role. Many common techniques for processing composites involve utilizing a vacuum bag or a difference in pressure in an autoclave to reduce the likelihood of voids in the finished product [19]. Printing nylon resulted in a reduction of porosity of 55% and an increase of tensile strength of 42%. It was postulated that the amount of heat generated by the printing process could be diminished under low-pressure conditions. This allowed for increased diffusion of molecules among the polymer layers, a thermal-driven process that improved bonding effectiveness and reduced residual stress [20].

## 2 Additive manufacturing technologies

Natural fiber-reinforced composite printing is an emerging technology offering an exciting new additive manufacturing approach. It combines the versatility of 3D printing with the eco-friendliness and sustainability of natural fibers. This innovative technique blends natural fibers such as flax, hemp, bamboo, or cotton with a matrix material, often made from biodegradable polymers like PLA. This results in a composite material that is both strong and lightweight while also being customizable and intricate due to the shaping capabilities of 3D printing. Natural fiber-reinforced composite printing represents a cutting-edge solution that benefits the environment and the manufacturing industry.

Additive manufacturing, also known as 3D printing, is a revolutionary technology that easily creates complex three-dimensional objects. The process involves using a digital machine to deposit materials layer by layer, gradually constructing the object. This technique is quickly gaining popularity as it provides several advantages over traditional machining techniques, such as milling, drilling, and cutting. With 3D printing, it is possible to fabricate polymer composites using a wide range of printing methods, creating intricate and sophisticated designs that were previously impossible to produce.

While some methods, including selective laser sintering, fused deposition modeling, inkjet 3D printing, stereolithography, and 3D plotting, are extensively used and have been around for a while, others are either in the experimental stages or exclusively employed by niche research communities. When it comes to fabricating composites, there are pros and cons to using each method. The production method is determined by processing speed, raw materials, resolution, product performance, and budget [21]. The ISO/ASTM 529000:2015 standard defines seven broad classes of AM techniques: Powder bed fusion (PBF), material extrusion (ME), vat photopolymerization (VP), binder jetting (BJ), material jetting (MJ), sheet lamination (SL), and directed energy deposition (DED) shown in Table 2 [22].

Different additive manufacturing methods use numerous readily accessible polymers such as powder, filaments, reactive monomers, and resins to create pure polymeric and composites made from polymers [23]. SLS, FFF, SLA, BJ, and DW methods are the most studied AM technologies for making polymer matrix composites. Many different AM techniques have been put into practice; these vary in their benefits and drawbacks based on factors such as part geometry/complexity, feedstock materials, and processing limits regarding resolution, and multi-material deposition.

### 2.1 Fused filament fabrication (FFF) method

The FFF process involves polymer thermoplastic filaments that are melted under pressure into a liquid and then deposited onto the build surface through a nozzle [24]. A CAD design has to be transformed into either an additive manufacturing format (AMF) or stereolithographic (STL) before it can be used during the AM process. The 3D layered model is then made. It is then built up from the ground up in stages. Programming language (G code) allows XY motion of the FFF extruder.

Most industrial FFF machines use specialized software for cutting and G-code generation. However, there are also circumstances when an STL model is sent straight to the FFF machine program. In the following step, known as “machine setup,” the values of the process parameters are given (such as build orientation, print speed, and infill density). Most FFF printers deposit layers by moving the extruder along a horizontal plane using a programmed tool path. The building platform moves down the z-axis when each successive layer is deposited [25]. As the design progresses, each new layer is constructed on top of the previous ones. The strength of the result relies heavily on the link among adjacent layers [26]. To allow adherence among the activated surface on the earlier deposited layer as well as the newly deposited layer, enough heat energy must be applied. The properties of the constructed models (such as their dimensional correctness, surface roughness, and mechanical qualities) are very sensitive to the FFF process settings [24]. This process is like that of extrusion or injection molding, except molds are not required. A heated chamber can reduce thermal distortion caused by uneven cooling. The FFF approach yields slightly greater anisotropics regarding characteristics compared to the SLA and SLS-based printing procedures. Crosslink production between and within FFF printed layers is one approach to mitigate the asymmetrical strength and ductility resulting from lower interlayer attachment than intra-layer strength in FFF printed layers [27]. Further, a detailed list of the process advantages and disadvantages of FFF is given in Table 1.

**Table 1:** Advantages and disadvantages of FFF.

Advantages	Disadvantages
1) An easy-to-understand manufacturing method that makes 3D printers.	1) Prolonged building times for highly geometrically complicated models.
2) The filament is more hygienic and simpler to swap out than alternatives that use powdered or liquid supplies.	2) The potential for minor surface flaws due to inadequate layer bonding.
3) 3D printers, in their assembled and kitted forms, and the materials and supplies needed to create these components, are incredibly inexpensive.	3) Inaccurate and low resolution when dealing with micron-sized components
4) The price of some 3D printers employing FDM technology is really low.	4) The filament's thickness determines the component's accuracy.
5) Use non-toxic materials, enabling equipment to be used even in office settings.	5) Lack of water resistance

**Table 2:** Categories of AM methods.

AM Category	Typical Materials	Max. individual cartesian dimensions of existing 3DP machines (mm)	Advantages	Disadvantages
Powder Bed Fusion (PBF)	Metals Ceramics Polymers Composites Hybrid	x ≤ 1400 y ≤ 1400 z ≤ 500	PBF's accuracy and precision in generating complex geometries make it ideal for sophisticated designs that might not be attainable with other manufacturing technologies. PBF uses only the material needed to build the product, reducing waste. This reduces material prices and environmental effects. PBF's design and object creation flexibility is ideal for one-off or limited-run production.	Expensive equipment Limited size Post-processing required. Safety concerns: PBF involves lasers or electron beams, which can pose safety risks if not used properly.
Vat Photopolymerization (VP)	Photopolymer (acrylates and epoxides) Ceramics (e.g., Zirconia, alumina)	x ≤ 2100 y ≤ 700 z ≤ 800	High resolution Low cost: VP can be more affordable than other 3D printing technologies, such as Selective Laser Sintering (SLS) or Stereolithography (SLA), especially for small to medium-sized parts. Speed: VP can produce parts faster than other 3D printing technologies, making it ideal for projects requiring a quick turnaround time.	VP is limited in terms of the size of parts it can produce. VP requires support structures to be printed with the part, which can be time-consuming to remove and can leave marks on the surface of the part. The resins used in VP can be hazardous and require proper handling and disposal procedures. Limited durability: The parts produced with VP can be brittle and may not have the same level of durability as parts produced with other 3D printing technologies.
Binder Jetting (BJ)	Polymers Ceramics Composites Metals Hybrids	x ≤ 4000 y ≤ 2000 z ≤ 1000	Large-scale production makes it a good choice for industries that require high-volume manufacturing. It is used with a wide range of materials, including metals, ceramics, and plastics. This technology allows for creating complex geometries, including internal structures, that would be difficult or impossible to produce using traditional manufacturing methods.	Produces parts with limited mechanical properties (inherent porosity due to limited solvent welding or chemical reaction bonding) Requires low viscosity ink Require significant post-processing (e.g., infiltration process)
Material Jetting (MJ)	Polymers Ceramics Composites Hybrids Biologicals	x ≤ 1000 y ≤ 800 z ≤ 500	High accuracy in droplet deposition. Low waste Multi-material and multicolor parts can be fabricated. The good surface finish of parts	Limited build volume Expensive Post-processing required. Material waste
Material Extrusion (ME)	Polymers Ceramics Composites Hybrids Biological	x ≤ 1005 y ≤ 1005 z ≤ 1005	Multi-material and multi-color parts can be fabricated. Inexpensive Easily scalable. Can build fully functional parts.	Poor surface quality Limited accuracy Limited strength Requires the use of support structures to prevent sagging or deformation during printing, which can be time-consuming and difficult to remove.
Direct Energy Deposition (DED)	Metals/metal hybrids	x ≤ 3000 y ≤ 3500 z ≤ 5000	DED is a fast process, capable of building parts quickly. DED produces parts with high density and strength, making it ideal for use in aerospace and other high-performance applications. DED can produce parts with intricate geometries and features, allowing for greater design freedom than traditional manufacturing processes. In-process monitoring: DED allows for in-process monitoring of the build, which enables real-time adjustments to the process, improving quality control.	Limited to metals and metal hybrids. A good balance between surface quality and print speed is required.
Sheet Lamination (SL)	Polymers Metals Ceramics Hybrids	x ≤ 250 y ≤ 220 z ≤ 145	Versatility Low cost High resolution Minimal waste: Sheet lamination can minimize material waste since it only uses the necessary material to create the part.	Limited strength Limited size To achieve a desired surface finish, the finished parts may require additional post-processing steps, such as sanding or polishing. Sheet lamination may have limitations on layer thickness, which can affect the printed part's final resolution and surface finish.

Additionally, fibers from nature were more widely used to reinforce polymers in FDM 3D printed polymer composites than synthetic fibers because their revival as reinforcement components for polymer composites correlated with their popularity in engineering uses.

Reinforcement for FDM printed composites often consists of synthetic fibers like glass, carbon, and Kevlar fiber. Graphene, carbon nanotubes, copper powder, powder, and so on are all potential synthetic fibers. In several published publications, FDM 3D printing with the addition of synthetic fibers to the matrix has been shown to improve the mechanical characteristics of polymer composites. Incorporating synthetic fibers into FDM 3D printed polymer composites improved or altered their thermal properties and conductivities.

**Fig. 2:**
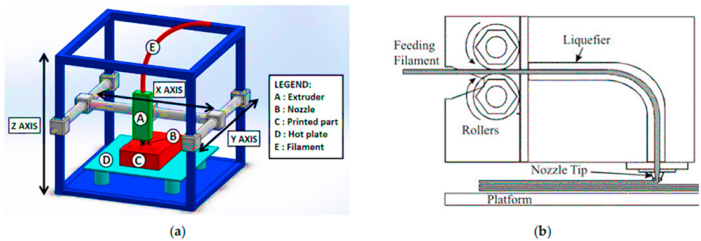
(a) A basic FDM configuration, depicted schematically (b) Filament deposition process and extrusion head design for fused deposition modeling (FDM), with authorization to reproduce and adapt from [26].

On the other hand, reinforcing thermoplastic composites using natural fibers improves their biodegradability and lowers their prices by reducing the amount of inorganic material used without sacrificing mechanical strength. Natural fibers such as wood, jute, bamboo, harakeke/flax, and sugarcane are frequently utilized in FDM 3D-printed polymer composites. The use of natural fiber-reinforced polymer composites as feeders in FDM-based 3D printing has been the subject of recent and thorough reviews. Because of its small equipment size, low cost, and capacity to create prototypes with complex geometries, FDM (Fig. 2(a and b)) is one of the most often utilized 3D printing technologies in pharmaceutical research. It also doesn't require chemical solvents [28]. Green engineering materials have been in the spotlight recently. The polymer composites made of organic polymers like PLA and soy-based resin bonded with natural fibers show tremendous potential because they are biodegradable and excellent for the environment [26]. Customized three-dimensional scaffolds can be made for specific patients by AM using a 3D scanner to create a specified CAD model of the injured organ [29].

#### 2.1.1 Fabrication of filament

The AM method produces NFRC filaments like regular filaments. However, preparation determines composite mechanical properties. Sifting, drying, and mixing impact the filament's quality and usability. How composites are manufactured affects RH interaction with the polymer matrix and load response. Drying and surface treatment improve fiber-polymer matrix bonding. Pre-mixing ensures uniformity, making the substance stronger. Thus, this section discusses current composite production methods before filament production methods. The process of making NFRC begins with sieving the fiber powder.

NFRCs' particle size must be decreased for AM, especially FDM, to avoid nozzle blockage. The powder is filtered to eliminate massive fragments from the composite, and the sieving process varies depending on the fiber source. In other words, it measures what makes for high-quality filament. Particles of 125 m in size caused the printer to jam while using a teak-based flour/PLA combination, but particles of 75 m worked well. Raw materials are dried in an oven to get the desired moisture content. This eliminates moisture in polymer granules and fibers. Materials with moisture may fail extrusion. If the polymer matrix has a high moisture content and the water evaporates during extrusion or hot mixing, gas bubbles and voids can occur. After extensive preparation, polymers and fibers create a polymer matrix. Researchers have used dry mixing, melt mixing, and filament extrusion. Before combining in polymer granules, a twin-screw extruder, fiber powder, and a compatibilizing reagent are air-dried and combined in an air-tight blender or by hand. Twin-screw extruders outperform single-screw ones when compounding. Due to their greater compounding ability, twin-screw extruders are employed in industrial extrusion applications that mix polymers with colors or fillers. FDM uses 1.75- or 2.85-mm filaments as feeders. FDM printers use software that presumes a fixed filament diameter, thus it's crucial that it stays consistent or only varies a little. The quality of a print might be damaged by using either too small or too large a filament.

Moreover, long fiber-reinforced polymer composites call for a uniquely shaped heating nozzle. Using an extruder, filaments of short polymer fibers are extruded and fed into a commercial heating nozzle. Numerous studies on short-fiber reinforcement have been prompted by this straightforward printing method. However, research into sintering/binding technology using powdered mixtures of polymers and natural fibers is scant. This occurs because of the uneven distribution of natural fibers and polymers throughout each layer due to their varying densities. When implementing gypsum sisal fiber powder in the binder jetting 3D printing process, a portion of the fiber diameter is larger than the layer thickness, leading to a higher level of porosity and a reduction in strength. A robust fiber-matrix contact was successfully generated through post-processing in this study. One study used fused filament production to strengthen a PLA matrix with jute fiber rovings. These composites' tensile strength and modulus were 5.11 GPa and 57.1 MPa, respectively, which are 157% and 134% greater than pure PLA, respectively. In contrast, the tensile strain was only 0.05%–0.2%. Uniform molding and enhanced mechanical qualities were cited as benefits that can be achieved with effective pre-tensioning of jute yarn fibers. Reinforcing polymer composites of hemp fibers with flax is common practice. These natural fibers had environmental benefits and helped make the composites stiffer than glass-fiber composites in tension and plate bending. As reinforcing additives, flax and hemp fibers are helpful in AM in fiber-oriented composites [30].

#### 2.1.2 NFRC filament production by using PLA

The processes involved in making the filament are the same as those used to make NFRC composite. The final phase, in which the composite is extruded into a long, thin thread, is the only one that varies from the norm. Creating an NFRC feeder for FDM is a straightforward process. Table 3 shows that attempts to make NFRC filaments out of polylactic acid (PLA) have been successful across all studied literature. This is because FDM is currently only performed with a small selection of polymers, including acrylonitrile butadiene (ABS), polylactic acid (PLA), and polyethylene terephthalate glycol-modified (PETG). Due to its favorable properties, such as a low melting temperature as well as low shrinkage, PLA has become one of the most prevalent polymers. Research on NFRC using polyethylene-based matrices of polymers for AM remains uncommon because of the challenges faced while using high-density polyethylene (HDPE) for FDM processes.

**Table 3:** AM's use of NFRC filament and the effect this has on mechanical performance [31].

Polymer Matrix	Fibers	Fiber Content (wt.%)	Results
PLA	Harakeke	0–30	At 20 wt% filler (36.8 MPa), Young's modulus ranges from 2.5 GPa to 4.2 GPa, resulting in the highest tensile strength.
	Hemp	0–30	Young's modulus raised from 2.5 GPa to 3 GPa; tensile strength peaked at 10 wt % filler (37 MPa) and decreased with increasing fiber content.
	Wood	40	The material's tensile strength dropped from 30 MPa to 10 MPa. From 80 MPa to 30 MPa of Flexural Stress.
PP	Hemp Harakeke	0–30	The average increase in tensile strength was up to 72% at 30 wt% filler, while the average increase in Young's modulus was up to 200% at 30 wt% filler. In contrast to empty PP.
	Hemp Harakeke	10–30	At 30 wt percent filler, tensile strength was increased by as much as 51 percent compared to unfilled PP.
ABS	Rice straw	0–15	Flexural stress at 30 wt % like unfilled ABS (50 MPa); overall tensile strength and modulus dropped from 35 MPa to 12 MPa.
	Lignin	0–40	Adding 40 wt% nitrile rubber improved the material's tensile strength overall.
HDPE	Cardboard dust	20, 50, 70	The incompatibility of the particles and the pure bonding ability resulted in worse mechanical qualities, such as lower tensile, bending, and compression strengths, compared to pure HDPE.
bioPE	TMP	10–20	With 20 wt% filler, the average tensile strength went raised from 10 MPa (unfilled) to 29 MPa. As fiber concentration rose, so did stiffness.

PLA = polylactide acid; PP = polypropylene; ABS = acrylonitrile butadiene styrene; HDPE = high-density polyethylene; PE = polyethylene; TMP = thermomechanical pulp.

It has been suggested that using a natural fiber powder with a particle size of less than 100 m can help prevent blockages. It has been reported that specimens with particles larger than 100 m were successfully printed. Composite printing was successful when 1 mm flaxseed fibers were used. It's important to note that even while researchers used a larger printer nozzle to lower the Polymers' probability of clogging and create composites that printed without a hitch, the print detail suffered as a result. The particle dimension range and associated nozzle dimension range that allowed for the successful printing of the composite are summarized in Table 4.

**Table 4:** Size Relationship Between Nozzles and fibre particles [31].

Nozzle Size	Particle Size
0.40 mm 0.50 mm 0.75 mm 1.00 mm	75 μm 100 μm 125 μm 1 mm (Length)

#### 2.1.3 Printing parameters

The yield strength of the substances, which impacts the quality of the print, is directly related to the nozzle temperature, according to recent studies on the effect of printing parameters on mechanical characteristics in the FDM process. As the temperature rises, the filament softens and bonds more strongly to the layer beneath it. For NFRC-based filaments, this is especially important because some fibers can only sustain temperatures of around 200 °C. According to the analysis, scientists typically print NFRC-based filaments at a nozzle temperature of 180 °C–210 °C. These days, you can find FDM printers with layer thicknesses as little as 0.05 mm (or 50 m). These incredibly thin layer heights may not be attainable in execution, however, because the particle size of NFRC filaments ranges from 75 nm to 125 nm. As a result, layer height estimates made with NFRC filaments now fall within a range of 0.1 mm–0.4 mm [31].

### 2.2 Selective laser sintering (SLS) method

The rapid development of SLS, which employs a semi-crystalline, particulate thermoplastic prepolymer, was quickly followed by SLA. Producing a component requires several different kinds of power. A bed of polymeric particles is preheated above their melting transition to recrystallize after cooling. As the powder bed is heated, less laser power is required for sintering [32]. Localized thermal sintering of the particles is achieved by tracing the 2D layer design with a powerful CO_2_ laser, which fuses the exposed particles within the layer and joins it to the previously scanned underlying layer. For efficient sintering without the physical and temporal overheating that degrades polymer quality, fine-tuning the laser power (up to 20–50W), beam size (usually 0.5 mm), beam speed, and distance between scans are necessary. A roller or blade deposits a 100-m-thick coating of prepolymer powder between sintering cycles. Polymer powders are 30–90 nm; therefore a coating is just 2–4 particles thick. Sintering requires less indirect heating. Particles must be permitted to travel across the layers because they are no longer compressed. Consequently, the thermal behavior, shape, particle size, and free packing density all play a role in the development of materials. Ground polymer processing is problematic because it produces an irregular particle form and a wide range of particle sizes, while spherical particles flow more readily and pack more densely.

**Fig. 3:**
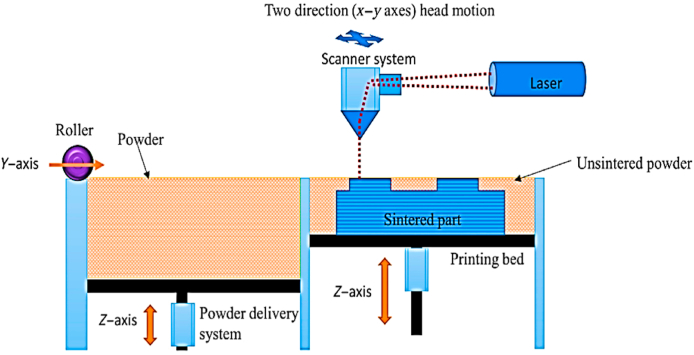
Schematic diagram of SLS [33].

Processing tiny particles with high cohesion or electrostatic repulsion causes problems. To strengthen, de-stress, and increase dimensional accuracy (which can be further improved by integrating shrinkage modeling in the initial part design), the building chamber is cooled at a low temperature after the object has been built [27]. With SLS, the powder is melted in whole or in part to create the desired object. Thermoplastics, metals, and even ceramics can all be printed using this method. Fig. 3 depicts the basic SLS operating principle.

In most cases, the apparatus is controlled by a laser of variable intensity [34]. The mirror reflects the laser beam onto the powder bed to create the layered effect. As the laser scans and warms the powder particles, they melt and fuse. After placing each powder layer, the roller and feeder mechanism lowers the building platform [35]. Printing any support structures is unnecessary because the unsintered powdered will hold up the hanging embellishments [33].

### 2.3 Stereolithography (SLA) method

**Fig. 4:**
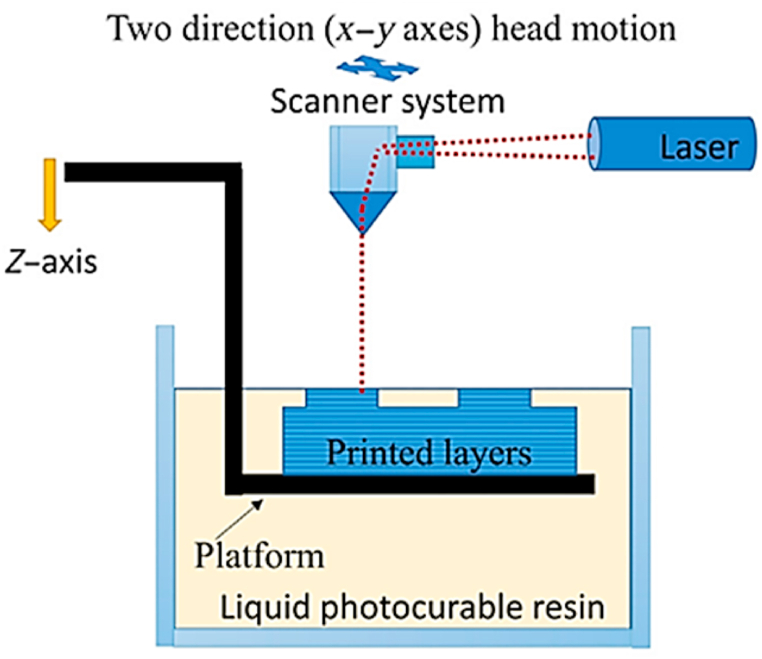
Annotated diagram of SLA [33].

The word “SLA” is now commonly used to refer to a variety of additive manufacturing techniques in which a liquid resin is exposed to a light source to selectively start the polymerization process of the material, resulting in a solid component [36]. The electronic current drives the laser light through the mirrors to the tank's base. During each layered iteration, the laser spot moves along a path determined by the program. The photosensitive resin is polymerized by applying light, which is repeated for each successive layer [37]. When compared to the hollowed pieces normally produced by conventional 3D printing technologies, the components produced by SLA 3D printing are solid. Micro-sized layers can be manufactured using lasers with spot sizes typically between 25 and 100 μm, creating structures with a smooth and uniform surface and structure. The capabilities of producing complex geometry structures are also improved by the SLA 3D printing process's specs. The medical, dental, and coherent fields make the highest use of SLA, followed by AM methods like DLP and LCD. While SLA technology is used for prototyping in industries like the automotive and aerospace industries, it is also put to use in the dental and medical fields, where the components are not only completely functional but also require precise fit and finish [38]. Since the total printing time for SLS and SLA is determined by the arrangement in a batch and scanned laser technology, the scanning time for a layer is typically substantially shorter than the recoating time (z-directional moving). To simplify the mathematical model, it was assumed that the print time for SLS and SLA (Fig. 4) technologies would be less than the print time for all parts [39].

### 2.4 Laser-guided direct writing (LGDW) method

**Fig. 5:**
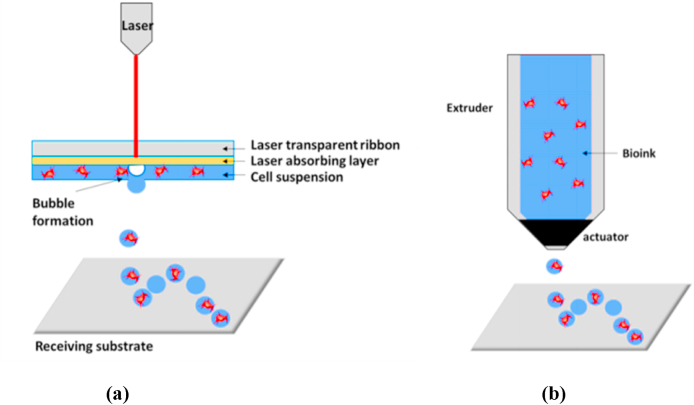
Representation of (a) Laser assisted bioprinting and (b) Inkjet Bioprinting [42].

LGDW is a laser-assisted bioprinting Fig. 5(a) process that enables micrometer-accurate cell deposition on various substrates and matrices, including soft gels. Hydrogel Inks, Gelatin-methacryloyl (GelMA), and other appropriate materials are used in the LGDW process. Concentrated laser beams can deposit cells onto different substrates and in different matrixes. With “laser-guided bio-printing,” cells are printed onto a substrate with the help of a laser beam. Inkjet printing allows for the distribution of droplets containing live cells with volumes between 1 and 100 pL. Continuous inkjet (CI) and drop-on-demand (DOD) are two methods for extruding droplets from a nozzle. In the field of tissue engineering, DOD is the top choice. DOD inkjet 3D bio-printing Fig. 5(b**)** typically employs droplets with 25–50 m diameter. When using CIJ for 3D bioprinting, a steady flow of 100 m-diameter droplets is released. Since the cell wall and viability are both affected by sonication at 15–25 Hz, electromagnetic and thermal inkjet printing have not been widely embraced in tissue engineering. This is why thermal inkjet is gaining popularity to improve cellular health. Many inkjet print heads are used to hasten the process, each equipped with many tiny nozzles. Bubbles occur when the ink is heated to a very high temperature, as in a thermal inkjet printer. The bubbles will keep growing until the ink is pushed through the nozzle. DNA, cells, tissues, and organs printed by this method remain viable even after being heated to temperatures of up to 300 °C for brief periods. In this instance, cell viability was estimated to be at 85% [40]. In addition, millimeter-scale cultures that can survive in culture for up to two months have been used to investigate heterotypic interactions between hepatocytes and endothelial cells, showing that deposition patterns can be programmed analogously to those of previous methods. Significant difficulties arise when using this technique in producing 3D objects due to the tiny size of cell arrays and the inability to stack cells in the Z-direction at present [41].

### 2.5 Binder jetting model

**Fig. 6:**
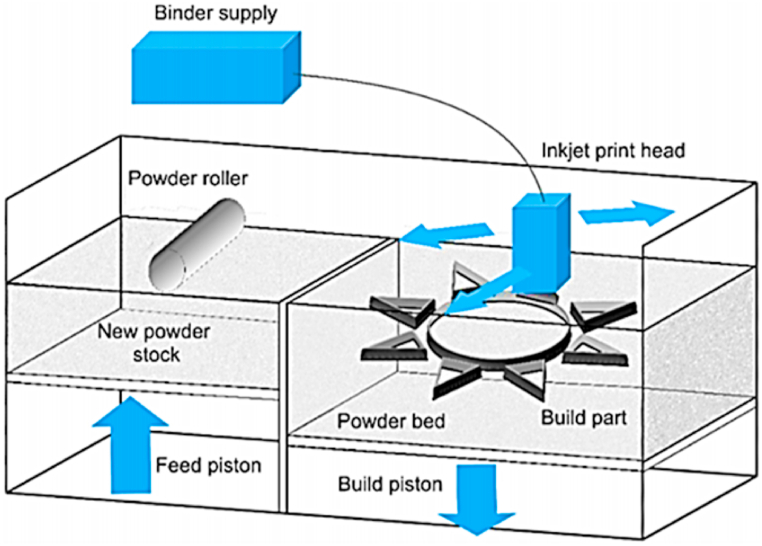
Schematic diagram of Binder jet [44].

Binder jetting (Fig. 6) is a type of additive manufacturing in which a bonding liquid is deposited selectively onto powder elements to create a solid three-dimensional object. With each subsequent platform drop, the print head adds more bonding agents to the powder, and the cycle continues. Among the many advantages of binder jetting are its inexpensive price, quick printing speed, big build volume, design freedom, and free support. Binder jetting can be used to print on various substrates, including metal, ceramic, glass, sand, and polymer. Silica sand, ceramic beads, stainless steel, chromite, zircon, bonded tungsten, soda lime glass, and tungsten carbide are all examples of high-quality commercially available materials used in industrial settings [43]. To achieve the appropriate density and strength, BJ-printed green objects must undergo a final step known as post-sintering in a high-temperature furnace, where the polymeric binder is burned off, and the powder particles are sintered together via atomic diffusion [44].

A concise overview of key aspects and features of current composite printing methods for future reference is shown in Table 5. The primary physical consequences during the process and the power source utilized are provided, together with the basic schematic for each technology. Even though FDM is now the most widely used AM method due to its low price and easy equipment setup, its major uses are constrained by the necessity for isotropic mechanical properties and detailed geometry, neither of which can be produced by FDM [45]. Due to the ultra-high resolution and surface smoothness, the SLA method is only used in niche applications, such as the manufacture of visual prototypes [46]. This technique can only be used for decorative purposes because of its inflexible limitations, including its material requirements and low mechanical performance. Powder and liquid bed printing techniques have their limitations when printing numerous materials at once with significant reinforcement [47]. Powder or nanofiller-reinforced composite printing is possible with practically all technologies and is therefore increasingly popular in AM to mitigate the process's drawbacks. SLS and 3DP can create short fiber composites with one-layer thickness. Only solid-based techniques can print long-fiber composites with a fixed fiber orientation [48].

**Table 5:** Comparison of AM technologies [[49], [50], [51], [52]].

Solid Grounded	Liquid Grounded	Powder Grounded	Hybrid – Powder Liquid Grounded
**Modern Technology**			
Laminated Object Manufacturing (LOM) Fused Deposition Modeling (FDM)	Polyjet Stereolithography (SLA)	Selective Laser Sintering (SLS) Laser Engineered Net Shaping (LENS) Selective Laser Melting (SLM)	Pro-metal Three-dimensional printing (3DP)
**Fundamental Regulatory Procedure**			
Adhesive binding for LOM Dissolving for FDM	Photo-polymerization, liquid deposition for Polyjet Photo-polymerization for SLA	Melting for LENS Sintering for SLS and SLM	Adhesive binding and sintering for Pro-metal Adhesive binding for 3DP
**Energy Supply**			
Laser beam for LOM Thermal energy for FDM	Ultraviolet laser for SLA Ultraviolet laser, thermal energy for poly jet	Laser beam for SLS and SLM Laser beam for LENS	Thermal energy for 3DP Thermal energy for Pro-metal
**Reinforcement**			
Powder, short fiber, and long fiber for both FDM and LOM	Powder for SLA	Powder, short fiber for SLS and SLM Powder for LENS	Powder, short fiber for 3DP Powder for Pro-metal
**Strengths**			
Low-cost printing on a variety of substrates requires no finishing work for FDM. When printing long fiber composites, there is a plethora of options for materials for LOM.	Excellent resolution and surface quality, no clogged nozzles, rapid printing, and no need for support structures for SLA. Quick printing and high-resolution multi-material printing for Polyjet.	It does not require a framework, can be made of various materials, and has excellent mechanical performance for SLS and SLM. On-site maintenance is ideal for fixing a broken component for LENS.	Many material options, no need for a support structure, and numerous binding permutations for 3DP. Many binding possibilities, but no underlying framework for Pro-metal.

## 3 Fiber reinforcement

### 3.1 Synthetic fiber reinforcement (carbon fiber/glass fiber/nylon fiber)

Artificial fibers have established a foothold in additive manufacturing ahead of their natural fiber counterparts. The literature reviewed found that synthetic fibers were the most effective reinforcing agent [30]. It has been discovered that the tensile strength of composite materials reinforced with synthetic fibers is greater than 100% greater than that of pure polymer counterparts. Short glass fiber, continuous carbon fiber, and short carbon fiber were used as reinforcing agents among other synthetic fibers. Both the modulus and tensile strength increased across the board in the reinforced composites. Excellent enhancement of characteristics in the filaments and final products can be achieved using synthetic reinforcements such as glass, polymer, carbon, nylon, and steel [53]. Nylon, a polyamide, was introduced in 1938 and used in wartime technology. Tenacity was 0.5 N/tex, and modulus was 2.5 N/tex. European and other tensile surface structures employ polyester. Geosynthetics also uses nylons like polypropylene. Polypropylene also makes drywall anti-crack products. Scientists developed stronger and stiffer high-performance fibers in the late 20th century [54]. Strength and stiffness are given the highest priority throughout carbon fiber production, although other properties like thermal expansion coefficient, electrical conductivity, and chemical composition might differ [55]. Physical qualities depend on carbonization level, stacked carbon plane orientation, and crystallization maturity. They better withstand fatigue, creep, and thermal expansion than glass or aramid fibers. This material has many advantages over conventional steel, including rigidity and corrosion resistance [56]. Structural engineering favors CFRP because it can rebuild aged bridges affordably.

Polymer nanocomposites have benefited from incorporating carbon-based nanofillers like graphene, expanded graphite, carbon black, carbon nanofibers, and carbon nanotubes. These fillers are easily recyclable and don't add much weight. Carbon nanofibers and nanotubes are the building blocks of a new class of materials called nanocomposites. Carbon fillers and polymer composites have been the subject of many studies, although it is still unknown how they will affect the final product's quality. Carbon nanotubes and graphite have become increasingly popular fillers in polymer matrices to improve composites' thermal and electrical conductivities, making them one of the most intriguing carbonaceous substances discovered in recent decades. Carbon nanotubes and other nanoscale structures are the materials of the 21st century. Incorporating carbon nanotubes into innovative polymer composites holds great promise for developing new materials with exceptional mechanical and electrical properties [57].

Glass-fiber-reinforced polymer (GFRP) provides several benefits over metals. Fiberglass is a strong, lightweight, and malleable material. Epoxy, thermosetting plastic (often polyester or vinyl ester), or thermoplastic can all be used as the plastic matrix. Fiberglass is frequently used for exterior door skins, roofs, pipes, water tanks, vehicles, bathtubs, hot tubs, and shower stalls. The thermal and mechanical properties of GFRPs are superior. Yet, environmental concerns have greatly complicated the recycling of GFRPs and reduced their use around the world [57]. Glass fibers add stiffness to composite materials, making them stronger. Glass fibers are weaker than aramids (by weight) due to their low moduli (35 N/tex) and tensile strengths (1.6 N/tex). High-temperature performance in metal and ceramic matrix composites for engines is where ceramic fibers shine. Their moduli can be greater than 100 N/tex (in terms of weight) despite their low tenacities of 1 N/tex. Mainstream glass fibers include E-glass, C-glass, and S-glass. While S-glass is more robust, E-glass is employed in electrical applications. C-glass is commonly used in the construction industry for making corrosion-proof parts. Civil engineers use E-glass to strengthen structures. Indoors and out, glass fiber is used for ceiling panels, floors, walls, curtains, and dividers [58]. Because long glass fibers can carry information, the matrix can be used for communication. Processing vapor-grown carbon fibers (VGCF) by mixed shear aimed to align the fibers and prevent fiber breaking. VGCFs have excellent rigidity and strength without sacrificing weight [59]. While carbon and glass fibers are typically used to strengthen composites, basalt fibers offer superior physical and mechanical qualities at a significantly lower cost.

The automotive, aerospace, and naval architectural industries all rely heavily on fiber-reinforced geopolymer matrices for various reasons. Steel, glass, and carbon fibers are synthetic fibers frequently used in geopolymer reinforcement. Carbon fiber reinforced plastics composite structures are widely used in the aerospace industry because of their high strength, specific stiffness, resistance, corrosion, and fatigue performance. Glass fiber augmented polymer composites, on the other hand, are being used in a growing number of 3D printing applications due to their low cost and exceptional performance. Glass fiber, as a material, has a high heat conductivity and a low coefficient of thermal expansion. The high curing temperatures required in industrial manufacturing won't harm fiberglass, making it a great material for 3D printing [3]. Polymeric fibers, including polyvinyl alcohol fibers and polypropylene, have been the subject of much research into their potential uses. Adding polypropylene fiber (PP) at 0%, 0.5%, and 1% of the total volume of a geopolymer mortar was recently studied. The mortars were dosed with 0.8, 0.9, and 1.0 silica modules at 12, 14, and 16 M M ratios [60]. Synthetic biodegradable polymers include polyesters, polylactic acid, and polycarbonate; synthetic non-biodegradable polymers include polyethylene and polypropylene [61]. A diamond-acrylonitrile butadiene styrene (ABS) composite filament was made by combining HPHT synthetic micro diamonds with ABS as a filler. Carbon fiber-graphene oxide (CF-GO) polymer composite resin and a mild annealing post-process have been used to create oct-truss (OCT) lattices [62]. Moreover, there is an ever-increasing need for both carbon fiber and Polylactic acid (PLA); researchers propose developing a recycled composite from various carbon fiber scraps to help combat the latter's ever-increasing waste [63].

Several studies have examined the feasibility of using continuous fibers to reinforce 3D-printed components [30]. This study developed a method for printing PLA composites with continuous carbon fibers that achieved tensile strengths of up to 190 MPa and flexure strengths of up to 133 MPa using a custom-built print head. Compared to the PLA-only specimen, their respective strength values increased by 4 and 3 [64].

On the contrary, many environmental issues, such as soil and water pollution, occupation of farmed land, and toxicities of live animals and plants, have been attributed to waste disposal from nonbiodegradable polymer matrices used in these composites. Mass production of these environmentally unfriendly materials contributes to depleting fossil fuel reserves and rising carbon emissions because they are made from polymers derived from nonrenewable resources like crude oil. In addition, the complex structures of waste polymer matrix composites make recycling them exceptionally challenging. After only two or three recycling, the quality of the material begins to degrade, making recycling impossible after that point. Cost-effectively, getting rid of this trash means either burning it for energy or dumping it in a landfill, both of which are bad for the environment. Matrix removal is a challenging process, making it difficult to reclaim or recycle the reinforcing fibers bound by the polymer [65].

Table 6 displays reliable statistics on synthetic polymer composites compiled from various credible sources. Here, the tensile and flexural properties vary due to the treatment and modification of the fibers used by various researchers. Researchers also tinkered with the matrix, changing the characteristics of both the matrix and the resulting composite.

**Table 6:** Composites bonded with synthetic fibers and their mechanical characteristics [57].

Polymer	Fiber	Strength		Modulus	
		Flexural (MPa)	Tensile (MPa)	Flexural (MPa)	Tensile (MPa)
Polyester	Kevlar Nylon Glass Carbon	95 – 16–444 –	159 – 60–85 –	2.2 – 18.2 –	8.7 – 72 –
PLA	Kevlar Nylon Glass Carbon	– – – 59–275	– 30–55 – 80–250	– – – 13	– 2–4 – 20
PP	Kevlar Nylon Glass Carbon	– 37–45 85 –	20–47 30–35 40–90 32–37	– 1.0–1.3 13 –	0.7–2.3 1.3–1.5 2–7.5 1–1.2
Nylon	Glass Carbon Kevlar	196–165 250 106–125	156–212 198 110–161	3.8–4 13.0 4.5–6.6	3–5 8.5 4.2–4.8
Epoxy	Kevlar Nylon Glass Carbon	160–250 42–48 297 550–850	300–375 7–18 179–784 20–100	3–6 – – 4–72	16–22 0.7 6.7–43 6–70
LPDE	Kevlar Nylon Glass Carbon	70–99 – 15–27 –	1500–1600 – 45–90 12–30	1.5–2.3 – – –	85–92 −1.5–2.5 0.22–0.41

### 3.2 Natural fiber reinforcements

Synthetic or biological resin can be used with natural or synthetic fibers to produce natural fiber composites. A “bioresin” is a “biodegradable resin,” a shortened form of the full term. Natural fiber composites' characteristic (tensile strength) is influenced by the resin type, fiber type, and manufacturing method applied. Fiber fraction and treatment also shape the features of composites made from natural fibers [66]. The lack of density and sustainability of natural fiber composites make them a desirable alternative to more conventional composites. These composites are gaining in popularity due to their non-carcinogenic and biodegradable properties. Compared to glass fiber and other synthetic fiber-reinforced composite materials, natural fibers have low density, high specific strength and modulus, less weight, high affinity, and biodegradability. For these reasons, they have been identified as crucial components for the growth of a green and sustainable economy. The primary constituents of natural fibers, or their derivatives, are cellulose, hemicellulose, and lignin. These renewable resources can be utilized similarly to strengthen, modify, and fill polymer matrices to create composite materials through 3D printing. Natural fiber composites are cost-effective for many applications, including warehousing, architecture and construction, packaging, and automobile and train interiors. These composites may find widespread use in place of more expensive glass fiber in light-duty structural applications. The exceptional mechanical characteristics of fibers can be utilized as reinforcing phases in polymer matrices or directly as printing inks, improving the mechanical performance of printed composite materials. These superior qualities encourage using 3D-printed fiber composite materials in biomedical applications. Natural fiber-reinforced 3D printing technology has yielded a wide range of sophisticated applications, such as antibacterial and mechanically stable medical devices, biomass inks with low cytotoxicity and high cell compatibility for tissue engineering, good bacteriostasis, high cross-linking strength, and enough mechanical strength to meet wound dressing requirements, freeze-dried hydrogel to create a 3D porous scaffold structure with high specific surface area and porosity, appropriate for drug delivery, support material for electrothermal conversion elements and lithium-ion battery electrodes, and electronic printing ink viscosity regulator. These composites are gaining in popularity due to their non-carcinogenic and biodegradable properties. Natural fiber composites are cost-effective for many applications, including warehousing, architecture and construction, packaging, and automobile and train interiors. These composites may find widespread use in place of more expensive glass fiber in light-duty structural applications. Natural fibers' low density, low cost, and biodegradability are all major pluses. However, one of the significant limitations of the natural fiber composite is its relatively high moisture absorption [67].

**Fig. 7:**
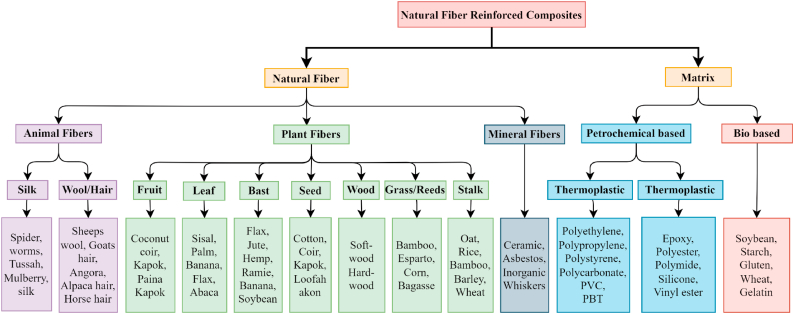
Classification of Natural fibers and matrices [69].

Natural fiber-reinforced composites (NFRC) were developed as part of the drive to increase plastic recycling and reduce plastic production. “natural fiber polymer composites” (NFPC) refers to materials encasing high-strength natural fibers, including oil palm, jute, kenaf, sisal, and flax in a polymer matrix. The polymer matrix employed can determine whether the final product is biodegradable or has dramatically improved mechanical properties at a reduced cost [31]. Plants, animals, and minerals are the sources of natural fibers. Feathers, silk, and wool are animal-derived natural fibers [68]. Several plants from which fibers can be sourced are Hemp (Cannabis sativa), Sisal (Agave sisalana), Coconut (Cocos nucifera), Bamboo, Kenaf (Hibiscus cannabinus), Flax (Linum usitatissimum), Ramie (Boehmeria nivea) and Jute (Corhorus capsularis). Based on their origin, the fibers can be classified as either bast fibers (flax, jute, ramie, hemp, kenaf), leaf fibers (sisal, banana, pineapple, and agave), seed fibers (cotton, coir, and kapok), core fibers (hemp, kenaf, and jute), reed and grass (corn, wheat, and rice), or another type shown in Fig. 7 [66]. The cellulose found in nature makes up these fibers. Thermosetting and thermoplastic matrices can both be strengthened using natural fibers.

A composite structure's ability to support tensile loads is significantly influenced by its matrix. The composite's binding constituent, or matrix, is crucially important. The four main varieties that have been documented are polymeric, metallic, ceramic, and carbon matrices. Polymer matrices serve as the foundation for most composite materials utilized today. Polymers can be roughly divided into two categories: thermoplastics and thermosets.

Thermoplastic matrix materials have a molecular structure of one or two dimensions, so they become pliable at elevated temperatures and restore their original properties when cooled. As a matrix for natural fiber-reinforced composites, the following kinds of thermoplastics have been used.

• Normal polystyrene (PS)
• Chlorinated polyethylene (CPE)
• Polyvinyl chloride (PVC)
• Polypropylene (PP)
• Low-density polyethylene (LDPE)
• High-density polyethylene (HDPE)
• Recycled thermoplastics

Thermoplastics are the sole option for natural fiber-reinforced composites due to the low integration temperature of the fiber into the polymer matrix (230 °C). Polyethylene and polypropylene are two polyolefines that account for most of these [70].

On the other hand, Thermoset is a crosslinked polymer that is rigid and stiff and does not soften or mold when heated. Thermoset polymers benefit from this structure in their strength, modulus, and adaptability to different applications. Thermosets are not as flexible as thermoplastics or elastomers. Many different polymers have been used as matrices for natural fiber composites. The most common thermoset polymers include epoxy resins and other resins, including unsaturated polyester resins (which are used in fiberglass), Phenolic Epoxy, Vinyl Ester, Polyamide, and Novolac [70].

The properties and efficiency of NFPCs are susceptible to a wide range of external influences. The hydrophilic characteristics of the natural fiber and the fiber loading impact the final composite's qualities. Generally speaking, large fiber loading is required to attain preferable NFPC properties. It is evident that an increase in the fiber content of a material that is composite results in better tensile properties. The process parameters employed to create them strongly affect the characteristics and surface aspects of composites. The best characteristics of the manufactured composite can only be attained by meticulous process technique and parameter selection [71]. Fiber-reinforced polymer composites have a long lifespan thanks to the combined stiffness of the reinforcing fibers and the resin matrix. With the right bio-filler and some surface modification, its performance on biocomposites can be improved. Coating biocomposites with an environmental barrier has shown promise in preventing deterioration due to exposure to the elements, heat, and mechanical stress [72].

However, the fiber's cellulose concentration and crystalline form alter as the source of the natural fiber shifts from leaves, stems, and fruits to agricultural waste [73]. Cellulose (60–80%), hemicellulose (5–10%), and lignin (10%) make up the bulk of plant fiber (five to ten percent). Other components include the very variable wax, tannin, humidity (up to 20%), and solvent chemical compounds. In addition to environmental benefits, there is a financial case for switching from synthetic to natural fibers because manufacturing synthetic fibers requires a lot of energy, whereas growing and harvesting natural fibers does not [60].

The researchers focused on how different fiber surface changes and production methods could enhance fiber/polymer compatibility. In contrast, several experts have studied and evaluated different kinds of natural fiber composites for their durability. Keratin, asbestos, and cellulose fibers are three of the most well-known types of natural fibers [74].

#### 3.2.1 Lignocellulosic fibers

Plants and trees produce cellulose (Table 7), also known as glucose polymers, as part of their normal processes. Even while all cellulose fibers are organic, their sources might vary depending on factors like regeneration. In scientific literature, cellulose-based fibers are described as polar and hydrophilic, and their interfacial adhesion and absorption are linked to their compatibility with cementitious and geopolymer matrices. This contributes significantly to natural fibers' high moisture absorption and is hence an important characteristic of these materials. The high moisture absorption of the fiber is improved from its original form when the cellulose microfibril is bonded together following the surface treatment process. Using natural fibers as textiles or reinforcements in geopolymer composites can be constrained by their hygroscopic nature. Studies have investigated the kinetics of water vapor sorption on filter paper, cotton, linen, jute, hemp, and sisal fibers using a dynamic vapor sorption device and analyzed the data using a parallel exponential kinetic model (EKM).

**Table 7:** Cellulose Fiber Types and examples [75].

Types						
Stalk	Grass/Reed	Fruit	Wood	Leaf	Bast	Seed
Examples						
Wheat, maize, oats, rice	Bamboo, corn	Coir	Hardwood, softwood	Abaca, banana, pineapple, sisal	Flax, hemp, jute, kenaf, ramie	Cotton

Lignin, a complex organic polymer, exhibits exceptional thermal stability compared to other cellulosic fiber components such as α-cellulose and hemicellulose. This is due to its highly cross-linked and aromatic structure. The order of thermal stability of lignocellulosic fibers is as follows: lignin > α-cellulose > hemicellulose. Although lignin starts to decompose at a temperature lower than cellulose (typically between 160 and 175 °C), it decomposes slowly over a wide range of temperatures, eventually extending as high as 900 °C. The temperature range of lignin's decomposition is broader than that of the other components of the fiber. Due to the high lignin content, lignocellulosic fibers generally produce significant residue during pyrolysis. Studies have shown that COIR and pineapple leaf fibers (PALF) treated with 4 wt% NaOH possess a higher residual mass than untreated fibers. The slow rate of chemical decomposition reaction and the domination of the formation of condensed cellulose as the solid residue is responsible for this outcome.

#### 3.2.2 Keratin fibers

Keratin is a fibrous structural protein that makes up the horns, hair, feathers, hooves, and claws of animals. Table 8 provides a quick reference to some of the most common animal fibers.

**Table 8:** Keratin Fiber Types and examples [74].

Types		
Hair	Hair Wool	Silk
Examples		
Horse, Camel	Sheep, Alpaca, Angora Rabbit, American Bison, Cashmere Goat, Mohair (Angora Sheep), Muskox	Spider silk, Silkworm silk

#### 3.2.3 Asbestos fibers

Asbestos is a material that occurs naturally and was widely employed due to its high resistance to heat in buildings and industry. In recent years, there have been considerable concerns that asbestos fibers could be present in additive manufacturing procedures like 3D printing. Materials used by some 3D printers, such as carbon fiber-reinforced thermoplastics, have been found to contain trace levels of asbestos, which makes their use undesirable. Additive manufacturing is not widely thought to provide a significant danger of asbestos exposure, yet it is still vital to take precautions. The release of asbestos fibers into the air can be avoided through cautious handling and disposal of asbestos-containing materials.

Table 9 shows two main types of asbestos fibers: serpentine (which is more coiled) and amphibole (which is straighter). Asbestos minerals fall into one of two categories: amphiboles, which include chrysotile, and serpentine, which exclusively includes chrysotile. Most chrysotile asbestos is found in structures built before 1980. Asbestos fibers are great for thermal and electrical insulation use because of their heat resistance.

**Table 9:** Asbestos Fiber Types [74].

Types	
Serpentine Asbestos	Amphiboles Asbestos
Examples	
Chrysotile	Actinolite, Amosite (asbestos grunerite), Crocidolite, Tremolite, Crocidolite, Anthophyllite

### 3.3 Pros, cons, and practical use of natural fiber

Some general qualities of natural fibers can be altered by processing, including their modulus of elasticity, tensile strength, and flexural strength. The main objective of fiber treatments is to increase the adherence of natural fibers to the matrix, hence enhancing the composites' interface. The type and amount of treatment chemicals can either enhance or impair the natural fiber's characteristics [66]. Table 10 displays various natural fibers' benefits, drawbacks, and uses. Devarajan mentioned that when continuous jute fibres were transformed into fibre filaments and coated with a plastic-based material for use as a 3D printing material, the resulting composites had a 28% increase in reliability at rupture but a 9% decrease in ultimate tensile strength. Jute fibre increased the breakage stress by 31% and decreased the bending stability by 35%. Additionally, they stated that studies on jute-based composites printed using additive manufacturing techniques are still in their infancy and that advances in printing and material preparation techniques could expand the uses of 3D printed biocomposites and reinforced composites made of jute fibre [76]. Priyadharshini declared that, Among the prominent areas where the digital touch has significantly improved final products are flaw detection in composites, composite material curing, autoclave manufacturing for composite materials, simulation for composite manufacturing, process control for composite manufacturing, composite manufacturing with AI and other digital techniques, and fibre layup. Thus, it is clear that using digital technology always makes the production process easier. It would be envisaged that fully AI-based tailored composite product development will occur soon. Artificial intelligence advancements in the production of composites with complex and customized shapes have the potential to expand the range of applications for composite materials [77].

**Table 10:** Natural fibers and their advantages, disadvantages, and application.

Reinforcing Fiber	Description	References
1) Coir	**Advantages:** a. Coir lasts longer than other natural fibers because of its high lignin concentration. b. When dry, coir fiber has very high interfacial adhesion. c. Coir fibers are superior to other reinforcement composites in efficiency and performance. d. Since coir is made from the fibres of coconut husks, it is a plentiful and renewable natural resource. Its application advances the creation of environmentally responsible and sustainable 3D printed goods. e. Since coir fibres have high strength and rigidness, they give 3D-printed parts exceptional mechanical qualities without incorporating much weight. f. Due to the low cost of coir, coir natural fibre composites are a feasible choice for 3D printing applications, particularly in areas where coir is easily accessible.	[67,71,78]
	**Disadvantages:** a. High moisture content b. It is less pliable because it has less cellulose than fibers like flax or cotton. c. Achieving consistent layer deposition and nozzle clogging are two potential problems that coir fibres may present throughout the 3D printing process.	
	**Application: P**aneling and roofing for buildings, vehicle interiors, packing peanuts, storage tanks, helmets, postboxes, paperweights, mirror casing, voltage stabilizer covers, and projector covers.	
2) Jute	**Advantages:** a. Affordable b. May be utilized extensively in the textile, woven, and nonwoven industries. c. Offers a strong high aspect ratio, strength-to-weight ratio, and good insulating properties. d. Because of the biodegradable nature of Jute, 3D printing employs it in a way that supports ecologically friendly and sustainable activities.	[67,79,80]
	**Disadvantages:** a. The resistance to creasing is quite minimal. b. Strength declines when wet. c. The mechanical and dimensional stability of 3D printed composites may be affected by jute fibres' propensity to absorb moisture from the surrounding air.	
	**Application:** For doors, furniture, windows, I-shaped beams, corrugated sheets, trenchless rehabilitation of subterranean drainpipes and fake roofing, water pipelines, jute fiber reinforced polymer composite, and floor tiles have been tested.	
3) Kenaf	**Advantages:** a. Swiftly expanding b. High yield of fiber c. Inexpensive and readily accessible d. Relatively little specific weight, excellent tension performance e. Kenaf fibres have excellent acoustic and thermal insulation qualities, expanding the spectrum of uses for 3D printed products.	[[79], [80], [81]]
	**Disadvantages:** a. Core sections absorb moisture at a comparatively high rate. b. It is challenging to handle and process long fiber bundles. c. It is difficult to apply binders to large fiber bundle lengths. d. High needs of water for growth e. The inherent fluctuations in the fibres make it challenging to print consistently and uniformly.	
	**Application: B**ags, Mobile cases, Clothing-grade cloth, insulation, packaging material, soilless potting mixture, and animal bedding.	
4) Hemp	**Advantages:** a. Very powerful and doesn't need pesticides. b. Less fertilization is needed, and it develops more quickly than other natural fibers. c. Fairly resistant to light frost and drought d. Due to their low heat conductivity, hemp fibres are suitable for insulation in printed goods.	[79,81]
	**Disadvantages:** a. The process of separating the fibers from the base requires a lot of labor. b. Limitations on cultivation and growth in several nations c. A lot of consideration may be needed to optimize processing parameters and guarantee compatibility with different 3D printing processes.	
	**Application:** Products for building, textiles, geotextiles, paper and packaging, furniture, electrical, note production, and pipe production	
5) Sisal	**Advantages:** a. Strong specific strength and simple accessibility b. Favorable acoustic characteristics c. Because of the high strength and endurance, sisal fibres are well-known for improving the mechanical characteristics of 3D printed goods. d. Low density of sisal fibers offer lightweight property in 3D printed goods.	[82]
	**Disadvantages:** a. Maximum processing temperature is restricted. b. It may be necessary to adjust processing methods and machinery in order to use sisal fibres with some 3D printing technologies, as opposed to synthetic alternatives.	
	**Application:** Panels, doors, closing plates, roofing sheets, paper, and pulp.	
6) Flax	**Advantages:** a. Relatively powerful b. Could be used to create garments c. Flax composites can give 3D printed things an aesthetically acceptable alternative because to their naturally beautiful appearance. d. Compared to other synthetic alternatives, flax fibre production usually produces less carbon emissions, which lowers the printed material's total carbon footprint.	[[83], [84], [85], [86]]
	**Disadvantages:** a. The initial stages of isolation are accompanied by significant dust production. b. Lack of flexibility c. When compared to certain synthetic substitutes, flax fibres could be less abrasion resistant. This property might raise questions in 3D printing applications where resistance to wear and tear is crucial.	
	**Application:** Bicycle frames, windows, panels, decks, railings, fencing, tennis racquets, forks, seat posts, laptop, and snowboard	
7) Ramie	**Advantages:** a. High strength b. Very high level of microbial resistance c. Hygienic qualities d. Because ramie fibres frequently exhibit thermal solid stability, 3D printed items can tolerate greater temperatures without a noticeable mechanical integrity loss. e. Ramie fibres are appropriate for applications where wear resistance is crucial because of their strong abrasion resistance. In adverse circumstances, this characteristic can help 3D printed items last longer.	[81,87]
	**Disadvantages:** a. Degumming is a challenge b. Energy-intensive treatments may be necessary to transform natural fibres like ramie into forms appropriate for 3D printing, impacting the material's total environmental impact.	
	**Application:** Filter cloth, sewing thread, and fishing nets	
8) Banana	**Advantages:** a. Satisfactory mechanical properties b. Having a high tensile strength, banana fibres help 3D printed objects by adding reinforcement. This strength may improve the printed components' mechanical qualities, making them appropriate for uses where durability is required.	[82]
	**Disadvantages:** a. The fibers cannot be consistently extracted methodically. b. The moisture absorption propensity of banana fibres might cause dimensional alterations in things that are 3D printed. This sensitivity could be problematic in situations where it's essential to maintain constant dimensions.	
	**Application:** Handcrafted papers, weaved fabrics, ropes, and mats could serve as an alternative to composites made entirely of glass fiber.	
9) Abaca	**Advantages:** a. Resistant to seawater and durable b. Appropriate stiffness, low density of abaca fibre offers light weight while maintaining high strength of the 3Dprinted objects. c. Recyclable, renewability, high dispersibility, and biodegradable properties are offered to the printed objects	[88]
	**Disadvantages:** a. Insects, molds, and fungus easily harm printed objects using Abaca fiber. b. It is not chemically resistant, making it incompatible with 3D printing technology.	
	**Application:** Used as a raw material for the production of high-quality paper, hospital textiles, machinery filters, electric conductors, etc. Other uses include making tea bags, fishing nets, meat casings, and tea bags.	
10) Bamboo	**Advantages:** a. Highly available and environment-friendly b. It has a unique antibacterial property, elasticity, and moisture absorption. c. Bamboo fibres give 3D printed items strength and longevity because of their excellent strength-to-weight ratio. Because of this, bamboo composites can be used in situations where durability is crucial.	[88]
	**Disadvantages:** a. The expense of logging, storing, and transporting it is higher. b. Factors like as age, species, and processing techniques can affect the characteristics of bamboo fibres. Because of these variances, 3D printing consistency could be difficult to achieve.	
	**Application:** Used to manufacture bathroom products, home decor, hygiene products, etc	

### 3.4 Characteristics of organic fibers

#### 3.4.1 Mechanical properties

The mechanical properties of natural fibers are affected by a wide range of factors. The mechanical properties of a natural fiber material depend heavily on the lengths and widths of the individual fibers [67]. Natural fibers' mechanical characteristics will depend on several variables. The primary variables impacting mechanical performance are processing techniques used for fiber extraction, harvest duration, aspect ratio (length/diameter), interfacial strength, matrix choice, fiber orientation, fiber dispersion, porosity, and composite manufacturing process. Research has shown that the modulus of natural fibers diminishes as their diameter increases [68]. Universal Testing Machine is used to test a variety of mechanical qualities, including impact and tensile strength. Numerous factors affect a composite's performance level or activities, but just a few of them are as follows.

I. Fiber orientation,
II. Fiber physical characteristics,
III. Fiber strength,
IV. Fiber interfacial adhesion property, and many more.

The mechanical efficiency of NFPCs is dependent on the stress transfer function among the matrix and fiber at the interface. The mechanical properties of NFPCs are influenced by various factors, including the orientation of the fibers, their ability to absorb moisture, the presence of impurities, the fibers' physical attributes, and the percentage of the volume of the fibers [71]. Fibers' physic mechanical properties are listed in Table 11.

**Table 11:** Natural fibers' mechanical and physical qualities.

Fiber	Elongation (%)	Young's Modulus (GPa) × 10^2^	Specific Tensile Strength (MNm/kg) × 10^−3^	Tensile Strength (MPa) × 10^2^	Density (g/cm^3^)	Moisture Content (wt%)
Hemp	1.6	0.58–0.78	3.67–7.4	5.50–11.10	1.5	6.2–12
Abaca	2.00–8.00	0.08–0.20	2.67–6.53	4.00–9.80	1.5	5–10
Pineapple	1.00–3.00	0.82	1.13–11.15	1.70–16.72	1.5	11.8
Aramid	3.30–3.70	0.63–0.67	21.4–22.5	30–31.50	1.4	–
Kenaf	1.6	0.40–0.53	2.33–5.81	3.50–9.30	1.50–1.60	9–12
Flax	2.70–3.20	0.28	2.3–6.9	3.45–10.35	1.5	8–12
Sisal	2.0–2.50	0.09–0.22	3.41–4.23	5.11–6.35	1.5	10–22
Carbon	1.40–1.80	2.30–2.40	28.57	40	1.4	–
Coir	30	0.04–0.0 6	1.46	1.75	1.2	8
Bamboo	4.1	0.11–0.89	2.33–1.67	1.4–10.0	0.60–1.10	8.9
Henequen	3.00–4.70	0.10–0.16	3.07–4.14	4.30–5.80	1.4	–
S-glass	2.8	0.86	18	45.7	2.5	–
E-glass	2.5	0.7	8–14	20–35.00	2.5	–
Banana	5.3	0.34	2.63	3.55	1.35	8.7–12
Jute	1.50–1.80	0.27	3.03–5.94	3.93–7.73	1.3	12.5–13.7
Cotton	7.00–8.00	0.06–0.13	1.91–3.98	2.87–5.97	1.50–1.60	7.9–8.5
Ramie	3.60–3.80	0.61–1.28	2.67–6.25	4.00–9.38	1.5	7.5–17

#### 3.4.2 Chemical properties

When a substance experiences a chemical alternate or response, its feature or behavior may be demonstrated as one of its chemical properties. Fourier Transform Infrared Spectroscopy (FTIR), X-ray diffraction (XRD), Atomic Force Microscopy (AFM), and Transmission Electron Microscopy (TEM) are instruments frequently employed to measure chemical characteristics [89]. Hemicellulose, Cellulose, and lignin are the three most prevalent chemical constituents of common fibers. Several crucial components of standard fiber, waxes, pectin, and ash, make up only a small fraction of the total. Natural fiber's main structural element is cellulose. It is the organic polymer that is most prevalent on earth. Cellulose is tasteless and odorless. It's probably the most common type of polymer found in nature. Hemi cellulose, a plant polysaccharide with a smaller molecular weight than cellulose, is a vital structural element in vascular cellulose's supporting tissues. Hemicellulose is a component of the cell walls of plants. Complex organic multicellular plants and a few algae. Cell walls, particularly those of wood and bark, rely on lignin for their stiffness and resistance to decay. Structures of vascular plants and some algae rely heavily on lignin, a complex organic polymer [89]. Microfibrils of cellulose are embedded in a lignin and hemicellulose matrix to form NF. The lignocellulose cellular wall can be understood as a naturally occurring composite structure consisting of a variety of chemical composites arranged in a spiral pattern. Different NFs have different amounts of water, ash, pectin, lignin, cellulose, hemicellulose, lignin, wax, and hemicellulose, among others. Wax is a common component of natural fibers. Although it is only present in minute quantities, it plays a crucial function in enhancing the bonding properties of natural fibers and influencing other aspects, such as fire resistance [90]. Chemical compositions in fiber vary in percentage based on where the plant grew, how fast it grew, and what part of the plant was used. The cellulose content of a plant or plant fiber determines its durability and rigidity [91].

According to grooving (climate, soil features aging circumstances, single fiber chemical composition (cellulose, lignin, hemicelluloses, waxes, pectin, and other minors), extraction/processing method conditions, mechanical properties and physical of composites are affected [67]. The percentages of cellulose, lignin, and hemicellulose in various materials are tabulated below in Table 12.

**Table 12:** Natural fibers' chemical compositions.

Name of fiber	Hemi cellulose (wt %)	Lignin (wt %)	Cellulose (wt %)
Coir	20	42	37
Henequen	4–28	8–13.1	60–77.6
Abaca	20–25	7–13	56–63
Alfa	38.5	14.9	45.4
Jute	13.6–20.4	11.8–13	59–71.5
Banana	19	5	62–64
Betelnut	32.98	7.2	53.2
Hemp	14–22.4	3.7–13	57–77
Palm	–	11–29	60–65
Cotton	5.7	<2	82.7–90
Sisal	25.7	12.1	78
Piassava	25.8	45	28.6
Flax	18.6–21.6	2.2	71
Ramie	5–16.7	0.6–0.7	68.6–91
Curaua	9.9	7.5–11.1	70.7–73.6
Bamboo	30	5–31	26–65
Kenaf	8–13	21.5	45–57

#### 3.4.3 Biocompatibility

High-strength composites develop when polymers are reinforced with natural fibers; these composites are biodegradable, inexpensive, lightweight, and have a superior mechanical structure. Even while lignin, cellulose, and hemicellulose all start breaking down at temperatures above 0 °C, natural fibers start to decompose at temperatures as high as 240 °C. The thermal stability of fibers is determined by their structural components, such as lignin and hemicelluloses, and can be altered by increasing or eliminating their concentration. Chemical treatments can assist in achieving this. Natural fibers degrade quickly and provide the least amount of environmental impact, but synthetic fibers pollute the environment as they do [71]. Synthetic resins shield the fibers well, but because they are mostly made of petrochemicals, they are not completely biodegradable. Because bio-resin is biodegradable, it is better for the environment than synthetic resin made from petroleum. Polycaprolactone (PCL), Copolyester, poly (ethylene terephthalate), poly (ester amide), (PET)-modified, polyhydroxyalkanoates (PHA), polyglycolide (PGA), polyvinyl alcohol (PVOH), poly(lactic acid) (PLA), starch blends and starch and other blends are a few of the researched biodegradable resins [66].

Natural fibers contain chemical components like hemicellulose, cellulose, and lignin. The composite eventually loses weight through soil burial following an interaction between these chemical components and soil-dwelling microorganisms. Polyester degradation is caused by the hydrolysis of the polymer's backbone. The polyester chain is then destroyed by soil moisture, resulting in microscopic composite fragments. By degrading them, the soil's microorganisms may utilize them as a source of energy. Fibre addition increased the proportion of weight loss as soil burial exposure time increased. With adequate fiber loading between reinforcement fibers, the hybrid composite kept its optimum biodegradability features [92].

#### 3.4.4 Thermal properties of natural fibers

The thermal characteristics of fiber-reinforced composites made of polymers are significantly affected by the qualities of natural fiber. The use of differential scanning calorimetry (DSC), thermogravimetric analysis (TGA), and derivative thermogravimetry (TGA) are all useful tools for evaluating the NFRP's thermal behavior under controlled conditions. Examining thermal characteristics (DTG) also makes use of differential thermal analytical measurements like differential thermogravimetric analysis (DTG) and differential thermal analysis (DTA) [92]. Temperature plays a crucial role in the thermal degradation of the fiber and matrix throughout the NFRP construction process, particularly the temperature at which they cure for polymers with thermoset properties and the temperature of extrusion for thermoplastic polymers. The proper filler material enhances thermal stability, and the composite is more suited for high-temperature environments [93].

OPF is expected to be more thermally stable than flax and hemp fibers, while unprocessed OPF is more thermally secure than treated OPF. As temperatures increase, OPFs' heat capacity grows. Increases in fiber content reduce the flax/HDPE composites' thermal diffusivity, thermal conductivity, and specific heat. For several natural fibers, including flax and hemp, enzymatic treatment Surface and thermal properties can frequently be improved by using natural fibers. The processes that can enhance the thermal characteristics of the fibers are hemicellulose and pectinase. When fibers are chemically treated with NaOH, the surface topography, thermal stability, and tensile strength are all enhanced compared to when they are untreated. However, when EFB fibers are chemically treated with NaOH and boiling water, they have even better thermal characteristics than untreated fibers [71].

## 4 Hybrid composite

Hybridization combines the benefits that different fiber types possess individually due to their synergistic interactions while minimizing their less desired characteristics. It is judiciously employed to increase the composite materials' toughness. Fiber hybrid composites have a matrix and several fibers. A hybrid composite made from distinct fibers and resin may have the best properties of its constituent elements and less undesirable ones. The goal is usually to keep one fiber's benefits while reducing its downsides. Hybridization can also create new traits or synergies that neither parent material could have alone. Flexural strength, fatigue, open-hole tensile strength, impact resistance, and tensile strength offer synergistic effects. As fibers are finer and interact more, these synergies strengthen. Reduced low elongation fiber volume percentage strengthens synergy. First-order estimates show that high-elongation fibers do not modify low-elongation fiber-hybrid composites' longitudinal stress failure strain. Due to their different longitudinal thermal expansion coefficients, the two fiber types may develop thermal residual stresses. The synergistic effects on yield stress are usually attributed to fracture propagation changes. Fiber breaks determine the longitudinal tensile failure of a unidirectional composite. The stress that a broken fiber imparts to nearby fibers is localized. Fiber break clusters are more common when neighboring fibers accumulate stress, increasing their failure likelihood.

Laminating numerous layers of each composite material or tightly combining the fibers in a matrix creates hybrid fiber-reinforced composites. Fiber-hybrids can be intralayer, treadearn, or interlayer. This simple category has two exceptions: unidirectional-multidirectional fiber hybrids. A woven cloth with glass fibers in the warp and carbon fibers in the weft is available in retailers. Fiber-hybrid intralayer fabrics are examples. Second, we can co-weave a non-hybrid yarn with an intrayarn yarn. This approach outperforms non-hybrid composites in mechanical strength. In addition to the key properties of both main components, the composite has unique features. The “hybrid effect” is nowadays hybridization's biggest problem. The components of the composite worked in tandem to get this outcome. Consider mode, hybrid ratio, and fiber type. Mechanical qualities greatly affect hybrid natural composite aspect ratio and water absorption [72].

The fundamental benefit of natural fiber hybridization is the ability to obtain good strength equivalent to synthetic fiber. The natural fiber was strengthened with synthetic fiber to tailor the attributes to the individual's needs. Examples include carbon fiber composites with high tensile strength but low toughness. Carbon fiber is fused with natural fiber to prevent this. Adding flax fiber to the high-flexural-strength basalt and carbon fiber composite enhances its bending properties. Reinforcing the basalt fiber composites using flax fiber increases their impact and tensile strength. Three different PLA hybrid composites were made from kenaf, bamboo, and coir fibers. The composite made from all three fibers outperformed the bamboo-coir and kenaf-coir alternatives in terms of tensile strength. Combining jute with oil palm fiber gives the composites superior tensile and dynamic mechanical properties. Bamboo and glass fiber hybridized with polyester boost the composite's chemical resistance capability. Nonetheless, hybridization may negatively affect mechanical properties [93].

The weight ratio of the fibers to the resin in the hybrid composite materials is 30:70 and includes sisal, jute, and glass fibers. Jute-epoxy, sisal-epoxy, sisal-jute-epoxy, and sisal-glass-epoxy are four composite material combinations. Resin transfer molding, hand lay-up processes, compression molding, and reaction injection molding are used to manufacture hybrid composite. Pultrusion and filament winding processes are also included [94].

### 4.1 Additive mixed (nanomaterial, silica aerogel, etc.)

#### 4.1.1 Nanomaterial mixed

Modern polymer matrices use a wide range of reinforcing components, from extremely hard to delicate particles, to boost their mechanical characteristics. Nanoscale materials (nanoparticles) like these novel enhancers can easily blend with polymeric matrices to form nanocomposites. It has been established that nanoparticles such as carbon nanotubes, graphene, and nano clay improve stiffness, toughness, and tensile and flexural strength [95]. Despite being orders of magnitude larger than the nanomaterials, atomic particles have a substantial surface area relative to their volume. Nanocomposites combine the advantages of multiple nanoscale phases (0D, 1D, and 2D) into a bulk material like ceramic, metal, or polymer for enhanced performance. The properties of nanocomposites are affected by several factors, including the nanoscale dimensions, loading (percentage by weight, percentage by volume), degree of dispersion, shape, size, and orientation of the nanoscale second phase, and interactions between the matrix and the second phase of the nanocomposites. The term “nanomaterial” refers to anything produced at the nanoscale, including metals, silicates, metal oxides, polymers, and carbon. Graphene, fullerene, ZnO_2_, carbon black, Fe_2_O_3_, carbon nanotubes (SWCNT, CNT, MWCNT), Nano clays, CaCO_3_, nitrocellulose, rubber, and TiO_2_ are a few examples of nonmetallic based nanomaterials. Other than gold and silver, some metallic nanoparticles include iron, cobalt, tungsten, platinum, copper, and nickel. If the right application, technique, loadings, size, and type of nanomaterials are used, adding nanoparticles to composite materials typically improves their mechanical characteristics and fracture toughness. If not, it might cause damage and have an unfavorable impact on how the bulk material behaves [96].

Structural applications have significant potential when traditional fibers (such as carbon, glass, aramid, etc.) are combined with extra nano-phase reinforcement (such as carbon nanotubes). By altering the epoxy matrix at the nanoscale, new FRPs are created with enhanced matrix-dominated mechanical characteristics and anisotropic conductive properties [97]. FRCs with relevant physical and mechanical properties can be created using waste lignocellulosic material and mineral filler (nano-SiO_2_). All composites reinforced with lignocellulosic material generally have better mechanical characteristics when their nano-SiO_2_ content is higher. Pure HDPE samples have poorer mechanical qualities than composites with fibers [98]. Because of these enhanced properties, studies of nano-fibrillated cellulose composites are being conducted for applications in engineering and medicine. Since these fillers are practical, a favorable cost-to-benefit ratio is required as industrial technology moves forward [99].

#### 4.1.2 Silica mixed

The compressive strength of the composite increases as the silica concentration rises. Silica particles on the outermost layer of the glass fibers decrease the initiation and rate of crack propagation by increasing the interfacial area [100]. For several forms of thermal insulation, silica aerogels are primarily used. SiO2 aerogels are also easier to dispose of, nontoxic, and inflammable than most other materials on the market, providing additional physical and ecological benefits [101]. Adding SA at lower concentrations improves the relationship between the fibers and matrix in the composite, preventing deformation and increasing the composite's stress tolerance. Higher concentrations of SA agglomerate within the composite lower the polymer's ability to moisten the filler, promote the development of cracks, and cause failure at lower loads. This behavior demonstrates that adding nanoparticles to natural fiber-reinforced composites above a particular concentration may have an adverse effect rather than an advantageous one. Up to a specific SA concentration, impact strength rises. Nonetheless, diminishes as SA is added more [102].

## 5 Properties of composite materials

### 5.1 Physical properties

#### 5.1.1 Density

The selection of matrix constituents and reinforcing fibers and the production process are all closely related to the density attribute. Manufacturers may customize the density of 3D printed composites to match specific requirements by carefully choosing these components and optimizing printing parameters. This makes them adaptable solutions for a variety of applications. One of the most important factors affecting the overall performance and applicability of 3D printed composites is their density. As composites, by definition, combine various materials with different properties to achieve a desired combination of characteristics, 3D printed composites are frequently engineered to have less density than conventional materials, which adds to their lightweight nature. Reducing weight without sacrificing structural integrity is especially helpful in manufacturing, aerospace, and automotive industries, where lightweight components can result in increased maneuverability, improved fuel efficiency, and more overall efficiency.

The Archimedes principle can be used to calculate the density. Density rises with higher fiber content. Pure composites made of polyester have the smallest void fraction, and composites with additional reinforcement fibers have progressively larger vacancy fractions [103].

Natural fibers can hold air because of the lumens in their cellular structure. This may also explain why void increases along with fiber loading. In addition to fiber loading, other factors that affect the void fraction include limited heterogeneous form, packing ability, matrix compatibilization, and fiber diameters. The voids impact the 3d printed composites' mechanical characteristics, and more voids mean more water absorption. Carbon fiber-reinforced polymers (CRFP-4) contain the greatest vacancy fraction comparable to other composite materials, whereas robotic fiber placement (RFP-1) has the lowest.

#### 5.1.2 Water absorption

A crucial factor in determining a material's propensity to absorb humidity over time is its water absorption capacity in 3D printed composites. The nature of reinforcing fibres, the matrix material, and the general composition of the composite all affect how much water is absorbed. Natural fibres found in composite materials such as wood, hemp, or flax are more likely to absorb water than synthetic fibers. The mechanical characteristics, dimensional stability, and general performance of the composite material can all be impacted by water absorption. As a result, the ability to absorb water is essential in applications where exposure to damp or humid conditions is probable, particularly in outdoor or aquatic situations. Manufacturers need to carefully weigh the advantages of composite materials against their propensity to absorb water in order to guarantee the best possible performance in certain environmental circumstances.

Natural fibers work well as reinforcement in plastics. Natural fibers have many benefits, but one major negative is their susceptibility to dampness. Because of their high absorption rates, 3d printed composites using natural fiber reinforcement can lose strength when wet, bend, or bulge in the presence of water, and place additional stress on supporting structures. Damage to composite materials' mechanical qualities can result from these factors, including warping, buckling, increased susceptibility to microbial inhabitation, and freezing and thawing [71].

Fiber loading positively affected water absorption behavior for all composites up to 96 h. The possibility of reaching a stable state exists then. Water absorption tends to rise proportionally with fiber content in natural fiber composites. Water absorption is enhanced by the fiber content and the interstitial space between the fiber and the lumen, the resin, the shell wall, etc. Superior fiber-to-resin bonding reduces water absorption by reducing capillary action in 3d printing [103].

Weak interfacial bonds could result from moisture in the fiber, lowering the durability of the printed composite and potentially reducing its useful life as well. According to the study, fiber treatment greatly increased the 3d printed material's resistance to water [93]. The chemical treatment decreases the water's global diffusivity. Four different reagents, including maleic anhydride (MA), acetic anhydride (Ac) acrylic acid, and styrene (S) can be used to perform the chemical treatment (AA). There are other various types of chemicals used in the treatments, such as alkali, silane acrylation, maleated coupling agents, benzoylation, permanganate, acetylation grafting and acrylonitrile, stearic acid, isocyanate, peroxide, triazine, fatty acid derivate (oleoyl chloride), fungal and sodium chloride. Surface treatments used for natural fibers have two main purposes: to strengthen the bond between the fiber and matrix and to increase the printed composites' stress-carrying capabilities [71].

#### 5.1.3 Tribology properties

It is crucial to take into account the tribological characteristics of 3D printed composites, particularly for instances where parts are subjected to rolling, sliding, or other mechanical interactions. These characteristics significantly influence the material's durability, effectiveness, and general performance. The tribological behavior of 3D printed composites is affected by a number of variables, including lubrication, types of reinforcement, surface finish, resistance to water and temperature, load bearing capacity, etc. The tribological characteristics of the composite can be greatly impacted by the selection of the matrix material and the addition of lubricating additives. Lubrication extends the life and overall efficiency of components by lowering wear and friction between moving parts. The tribological behavior of the composite can also be influenced by the kind of reinforcing fibres used in it. For instance, adding carbon fibres or other specific fibres can improve the material's resistance to wear. A clean surface finish enhances the composite's overall tribological performance by lowering wear and friction. A component's ability to withstand substantial stresses depends on its 3D-printed composite's load-bearing capacity. Proper material selection, reinforcing, and design considerations are necessary for a material to sustain applied forces without experiencing undue wear. The tribological behavior of the composite may be impacted by applications that entail high temperatures. Important things to think about are thermal stability and resistance to fluctuations in temperature.

In moving relative to other materials, 3d printed composite materials are subject to wear and friction. The composite material suffers tribological damage as a result. Generally, tribological damage results in the material's surface degrading rapidly**.** The tribology performance could be developed by choosing the right reinforcement mix within the polymer matrix. A reinforced composite's wear rate is higher than a pure composite's. Evidence suggests that kenaf fiber reinforcement in epoxy composite significantly boosts wear performance by as much as 85% compared to that of pure epoxy composite. The fiber-reinforced treated composite has effective results against wear. The tribological characteristics of fibers or polymers can be changed (either favorably or negatively) through reinforcement. Incorporating natural fibers improves PLA's wear durability, and the wear rates of composites were noticeably lower than those of neat PLA under higher loads [71].

The wear rate behavior of 3d printed composites increases with fiber loading, as determined by the dry sand abrasion wear test. The printed composite's wear resistance can be greatly enhanced by increasing the material's hardness. The wear resistance of a material is determined by its hardness and flexibility. In terms of specific wear, pure (PP) composites are superior. Lower abrasion resistance due to more voids in the fiber reinforcement causes a greater specific wear rate. Fiber-reinforced composites have superior bonding adhesion between the fiber and matrix, therefore, wear rates do not rise with the amount of fiber loaded on during a wear test [103].

### 5.2 Mechanical properties

#### 5.2.1 Tensile properties

The direction of the reinforcing fibres inside it significantly influences the 3D printed composite's tensile strength. For best performance, proper fibre alignment and distribution are necessary. The composite's tensile strength is mostly determined by the kind of reinforcing fibres that were utilized. Natural fibres like hemp or flax, glass, carbon, and aramid fibres are examples of common reinforcing fibres. The unique tensile characteristics of each kind of fibre add to the composite's total strength. The fiber improves the composite's tensile qualities when mixed with polymer material. Fiber length is a controllable factor in NFRP, allowing for surface area and tensile properties modification. In general, composites' tensile strength is seen to increase linearly up to a certain threshold and subsequently decline in all matrix types [93]. Studies show that the bending properties and tensile of jute/PP (polypropylene) unidirectional composites, including only 20% jute fiber, are vastly superior to virgin polypropylene specimens. The tensile characteristics of the composites were shown to degrade after being subjected to water absorption tests at several temperatures [104]. The selection of the matrix material, which is usually a polymer or a polymer blend, also affects the tensile strength. The matrix material adds to the overall mechanical qualities of the composite and acts as a binder for the reinforcing fibres. The tensile strength of 3D printed composites can be affected by printing parameters including layer thickness, infill density, and printing temperature. To get the intended mechanical qualities, these parameters must be optimized.

Composite materials with 0, 10, 20, and 30% fiber by weight have been evaluated for tensile strength. Tensile strength is shown to be lower with a 10% fiber loading compared to a 0% loading (raw polyester). Possible causes include flawed fiber behavior at low fiber loading. The tensile strength is then improved by increasing the fiber loading. The increasing tensile strength is a result of the reinforcing action caused by the fibers. The fibers' strengthening effect allowed for uniform stress transfer from the polymer matrix to the fibers. A dispersed matrix has been generated as an additional attribution. Because of these occurrences, stress was distributed evenly throughout the material, which increased its tensile strength [92].

Improvements in strength at tensile stress with fiber loading may be attributable to the strong bond formed between the fiber and matrix. Rambans fiber, in both its chopped and unidirectional mat forms, normally exhibits increased tensile strength up to a certain limit of fiber loading; however, the inclusion of filler alters the tensile strength of 3d printed composites. This may be due to insufficient adhesion at the fiber/filler contact or poor particle dispersion, both of which hinder effective stress transfer. It was discovered that the tensile and flexural strengths of the bamboo fiber reinforcement 3d printed composite rose with fiber loading up to around 15 wt% but then began to decrease [103].

#### 5.2.2 Flexural strength

One of the most essential mechanical properties of 3D printed composites is their flexural strength, which measures how well the material can bear bending or flexural loads before breaking. This feature is especially crucial for applications where components such as structural elements or components under dynamic loading may be subject to bending or flexing forces. The flexural strength of a 3D printed composite is primarily determined by the distribution and orientation of its reinforcing fibres. Improved structural integrity results from the fibres being positioned and distributed correctly. When the fiber reinforcing is strong and doesn't break easily, the strength at tensile stress is higher than when the fiber-reinforced is flexible. The composite's homogeneous and heterogeneous properties determine whether or not this occurrence occurs. It examined the mechanical properties of a polyester matrix filled with Napier grass fibers. The fiber was reinforced in two unique ways: with short and long fiber. The flexural durability of the short-fiber reinforcement was higher than that of the long-fiber reinforcement [93].

The flexural strength was measured for the synthetic composites made with 0, 10, 20, and 30% of fibers. Flexural strength was seen to decrease as fiber loading increased. This lower outcome could have been brought on by bubbles that appeared throughout the production process. The contact with the natural fiber reinforcement and the polymer matrix is crucial to the overall performance of the composite. The prior study evaluated the mechanical properties of a polyester composite with rattan fiber reinforcement made using a hand lay-up technique. It rises dramatically with increased fiber loading up to a certain value. After that, it was lowered since there wasn't enough resin to support the fiber's weight. Another study claims that bamboo fiber was used as a reinforcing material in a polypropylene matrix to characterize mechanical properties at fiber loadings varying from 0% to 60%. Flexural strength was found to grow in tandem with fiber loading [92].

#### 5.2.3 Hardness

The hardness of the 3D printed composite is largely correlated with the fibre volume fraction and modulus. As the fibre weight fraction rises, the printed composite gets harder. The removal of lignin and hemicellulose increased hardness because it increased interface adhesion. The Vickers hardness values drastically decrease as fiber loading increases beyond a certain level. This could be a result of the small void gap that results from insufficient matrix resin and fiber bonding or wettability during composite production [103]. In situations where 3d printed components may be subjected to abrasive pressures or wear, hardness is especially important. Several variables, such as the variety and arrangement of reinforcing fibres, the matrix material, and the composite's general composition, affect how hard 3D printed composites are. The orientation and distribution of commonly used reinforcing fibres, such carbon, glass, or aramid, are important factors that affect the composite's hardness. The matrix material, which is usually a polymer, also affects the total hardness by giving the reinforcing components cohesion and support. The hardness property of 3D printed composites can be further improved with careful parameter and post-processing treatment optimization.

Homogeneous fiber dispersion in the matrix and robust interface bonds contribute to the printed composite's hardness. The distribution of fibers inside decreases the polyester matrix's flexibility. More stiffness and rigidity are produced in the composite. With increased fiber loading, the hardness of the composite likewise increases as its stiffness does. For the synthetic composites that contained 0, 10, 20, and 30% by weight of fibers, hardness was measured. The hardness rises when the fiber loading is improved. The polyester composite was reinforced with rattan fiber to describe the mechanical qualities. It was discovered that hardness rises as fiber loading does. The mechanical characteristics of a 3d printed composite made from rattan fibers and polypropylene were evaluated and contrasted to those made from glass fibers and polypropylene. High crystallinity was responsible for the increased toughness of rattan fiber-reinforced composite [92].

#### 5.2.4 Impact properties

Compared to its other properties, 3d printed composite has a relatively low impact strength; nevertheless, impact strength can be increased by adding fiber loading up to a certain limit due to the fiber's role as a load-transfer medium. Consequently, they are effective at halting the spread of cracks. The impact resistance of printed composites can be increased by including fiber in the matrix. As the percentage of fibers in 3d printed composites increases, so does the energy required to break down the bond between fibers and the matrix, which raises the risk of fiber pull-out [103]. Since 3d printed composites offer greater impact strength, they are advantageous for automotive applications and other uses that demand high levels of impact resistance. The impact strength of cotton-reinforced composites improved when adding epoxy or agro-based resin [66]**.**

Higher impact strength depends on the type of fiber and how it binds. Due to the fibers and matrix's favorable adhesion, it was discovered that the impact power increased as the number of fibers was increased. Micro-spaces are produced when the fiber and matrix do not adhere well. With impact loading, these micro-spaces easily cause microcracks. Impact strength may be reduced as a result. The impact strength was tested for the manufactured composites having 0, 10, 20, and 30 % by weight of fibers. As fiber weight content was increased, it was found that the impact strength also increased. The impact strength of natural fiber-reinforced polymer composites is influenced by the type of fiber and polymer matrix and the degree of adhesion between the fiber and matrix. The impact strength was improved because the fibers could tolerate energy thanks to the tight interfacial interaction between the fibers and the matrix. Impact damage to a composite may result in failure owing to fiber withdrawal. Rattan fiber was reinforced in a polypropylene matrix to characterize the mechanical properties. The impact strength was found to decrease with increasing fiber loading. The mechanical properties of a polypropylene matrix with bamboo fiber reinforcement were studied at varying fiber loadings. Since there were more fibers to produce stress transfer, the impact strength also increased with fiber loading [92]. It was discovered that jute's unidirectional fibre orientation has a better impact strength than successively woven fibre mats used in composite materials with alternate directions, as well as the incorporation of additional foreign elements to create hybrid composites [105]. The following Table 13 summarizes the typical properties of 3D printed composites compared to conventional composites.

**Table 13:** The typical properties of 3D printed composites compared to conventional composites.

Properties		3D printed composites compared to conventional composites
Mechanical	Fiber Volume	To ensure good adhesion between adjacent print beads and layers for improved structural integrity, the inherent nature of 3D printing requires a relatively larger amount of matrix material than traditional composite manufacturing technology. Examples of these processes include injection molding, resin transfer molding, and compression molding. It is rare to find 3DP composites with 40% Fiber Volume Fraction (FVF), whereas conventional composites can easily have more than 60% FVF.
	Tensile strength	After comparing the structures of unidirectional (UD) and 3D orthogonally woven (3DOW) composites, it was found that the 3DP composites had a higher tensile strength. The characteristics of the matrices and fibers used in the two composites can be used to explain why the 3DOW composites' matrix and fibers had a greater tensile strength. Furthermore, the 3DP composites' poor structural integrity and higher void content may be crucial in the initiation and spread of cracks, which could ultimately lead to the composites failing too soon.
	Interfacial bonding strength	Although the FDM approach has less printing pressure than in typical production processes, the interface influences would be more significant. When stacked continuous carbon fiber-reinforced layers (CCFRLs) are separated into composites, the mechanical characteristics of the materials are improved. This effect is explained by the initial interfaces of dispersed specimens adhering significantly more strongly than those of concentrated specimens. The interfacial bonding strength between printed layers significantly influences the mechanical properties of 3D printed composites. Printing settings significantly impacted the mechanical properties of continuous fiber-filled composites that were 3D printed. The mechanical properties of 3D-printed hybrid composites are contingent upon various factors, including the characteristics of individual reinforcing fibers, printing conditions, and the interplay between various printed filaments and layers.
Structural	Complex shape formation	Complex-shaped products are produced by material removal procedures in conventional composite manufacturing techniques, including casting, molding, and machining. Although composites' performance and manufacturing process in these technologies are well-understood and controlled, there is limited control over the intricate interior structure. Complex composite constructions can be created with 3D printing without the usual waste. With computer-aided design, composites' size and geometry may be accurately controlled. As a result, composite 3D printing achieves an excellent balance between process flexibility and high-performance outputs.

## 6 fiber-reinforced additive manufacturing (FRAM) in industry 4.0

**Fig. 8:**
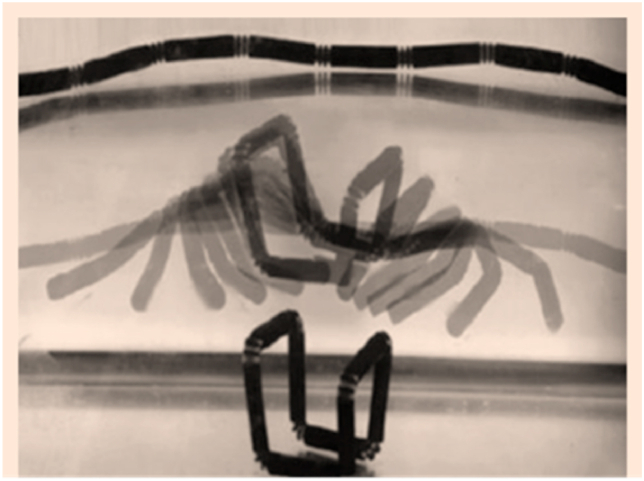
MIT has come up with a 3D printing process that can convert a 2D shape into a 3D one using water-absorbing polymers [107].

As a result of the related opportunities of FRPs in areas such as raw element choice and distribution, filler and additives addition, and material shape, it is anticipated that transitioning to Industry 4.0 will substantially boost productivity as well as flexibility metrics while simultaneously opening new design opportunities. Machine learning, AI, big data, 3D printing, and robots are examples of quickly developing technology directly affecting the Internet of Things (IoT). The IoT's ability to aid objects in sensing the environment around them gives such devices life, which is a key factor in the profound shifts that accompany such technological advances [53]. Evolved customized composite materials that respond to case-specific needs are easier to create and implement in robotic and additive technologies. Scenarios are constantly and swiftly changing; recent advances in the concept of AM have resulted in the definition of 4D printing procedures. These novel techniques are based on 3D printing of smart materials, the shape of which can be altered in real-time during the deposition process in response to external stimuli (such as electricity, heat, magnetism, etc.) and the use of the physical property evolution of resins (such as shrinking, softening and curing hardening) to advantage. Robotic additive systems carry out fiber deposition tactics and advanced shaping procedures to produce custom FRP blanks. Robotic additive systems' versatility in working with various materials, accurate fiber orientation, additive distribution, blank shaping, and control over polymer softening and hardening makes them ideal for producing FRPs. Robotic AM techniques in this category of materials facilitate the definition and creation of hybrid multi-material FRPs [106]. Material properties, such as the amount of light they absorb or reflect, or their thermal or mechanical stability, can change over time in smart materials. MIT researchers have recently introduced using materials with water absorption characteristics to create 4D printed structures, and they have printed multi-materials with the ability to alter shape underwater, as shown in Fig. 8.

As innovation and technology advance, 4D printing is becoming increasingly popular in the manufacturing and engineering sectors. The fields of aviation, automotive, healthcare, robotics, aerospace, defense, art, textiles, and so on are just a few of the many that benefit from 4D printing. The medical field is the primary user of 3D bioprinting to create living biological 3D structures like organs, tissues, nutrients, and cells due to its high accuracy in positioning the various types of cells, its potential for mass production of tissue-engineered products, and its ability to fabricate high cell density tissue. 4D printing, also known as laser-assisted bioprinting, is a relatively recent approach boasting superior resolution in biological printing. The bio-origami hydrogel scaffolds used in 4D bioprinting can self-fold and unfold in response to stimuli, and they fall under the category of “smart hydrogels,” which are printed in 3D.

In Industry 4.0, robotic and additive manufacturing are two crucial components considered for composite manufacturing. Constant improvement in our understanding of the issue is essential if we are to use innovative methods. Some possible future research directions that could be tailored to different applications are described below.

Process optimization is a key topic of study in robotic composite manufacturing. Optimization of specific processing parameters and limitation of associated flaws remains a significant problem despite the considerable progress documented in the literature regarding online path correction, mobility, and control. To get beyond these obstacles, the Industry 4.0 paradigm calls for intensive study into optimizing robotic layup processes.

The production of multi-material composites is another possible area of investigation. The multi-material AM technique has used fiber-reinforced polymer composites, allowing for the creation of heterogeneous structures for various scientific and technical purposes without limitations on geometric variation during the design stage. The application has additional potential in the field of 4D printing and with various material combinations. One of the newer avenues that is getting a lot of attention is 4D printing, which has the potential to improve printing processes, encapsulate smart materials, and find compatible printing materials. New research shows that continuous CF-reinforced shape memory polymer composites created via 4D printing have higher mechanical strength than 3D printed composites. Incorporating continuous CF into developing complicated components could form the basis of future research endeavors.

High-performance thermoplastics like PEEK and PA6 continue to garner a lot of attention when it comes to materials used in AFP processes. Industrial uses of AFP techniques are projected to shift away from traditional thermoset resin systems and toward these high-performance thermoplastics, which offer better mechanical qualities, are more quickly produced, and are recyclable. The detection of surface and interior flaws, including voids, overlaps, gaps, and fiber wrinkling, that are linked with automated manufacturing processes has been greatly improved in recent years through the effective use of many online defect-monitoring systems. With the help of these recently created online defect-monitoring systems, industrial-scale robotic layup processes can improve their defect mitigation capabilities, reducing costs and increasing production. Composite structures made using automated techniques are mostly used by the aerospace industry, which is seeing fast growth. The current industrial transformation is predicting the need for highly efficient, fully automated AFP machines with optimum processing parameters to meet the industrial demands of aerostructures. Significant advancements in automated composite production have already been spurred by Industry 4.0, and many more advancements are anticipated in the years to come.

Multiple thermoplastic resins and GF reinforcement combinations have been successfully FDM 3D printed using the in-melt simultaneous impregnation technique for continuous fiber-reinforced composites. The results regarding mechanical property variation were encouraging, so it would be wise to apply a similar strategy to different combinations of thermoplastic resin and CF reinforcement in the future to see how widely applicable this innovative scheme can be. It would be helpful to test the method's adaptability for various combinations using a strategy similar to that of other geometries, including curved surface printing. We should also focus on the difficulties caused by this method's mandated maximum fiber volume percentage of 50%.

7. Application of 3D printing with fiber-reinforced composite and nanocomposites.

### 6.1 Automotive and aerospace industries

**Fig. 9:**
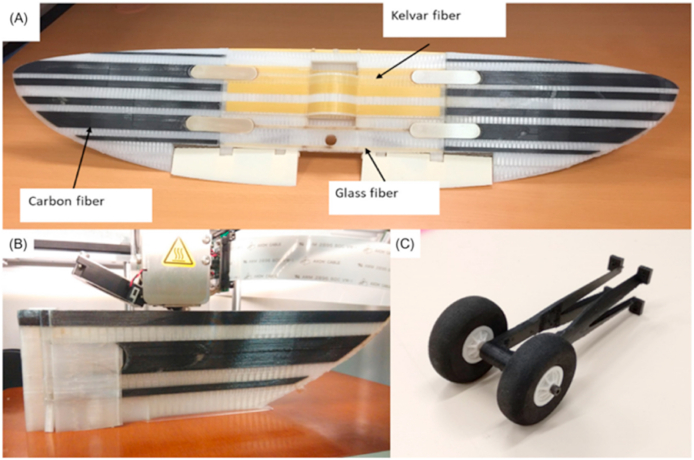
(A) a wing consisting of Kevlar, carbon, and glass fiber-reinforced thermoplastics; (B) uninterrupted carbon-fiber lamination on the UAV wing; and (C) the landing gear are all examples of components of an unmanned aerial vehicle (UAV) that were 3D printed [33].

The aerospace industry (Fig. 9(a–c)) has benefited greatly from the development of 3D printing technology. The technology provides a product with complicated engineering geometry that can be manufactured quickly. Products made using 3D printing technology are ideal for use in the aerospace sector because of their many benefits, including low manufacturing volumes, excellent quality, low weight, and resistance to high temperatures.

Fiber/Nano-reinforced composites 3D printing processes are outlined in Table 11. By incorporating continuous fibers into the 3D printing process, it is possible to create lightweight structures with a high strength-to-weight ratio, making the technology applicable to the aerospace and automotive sectors. Nanocomposites enhance composites' electrical and thermal conductivities and improve their delamination qualities. In Table 14, we can see multiple instances of electronic components in which 3D has been printed. In addition, 3D printing allows for the production of lightweight 3D cellular composites with qualities like low density and the possibility of multifunctionality, much like balsa wood [33].

**Table 14:** Fiber-reinforced composites and nanocomposites together are being implemented in the aerospace industries and automotive use of additive manufacturing.

Technique	Applications
Material extrusion	Bicycle frames, printable mold tooling, unmanned aerial vehicles (UAVs), tissue engineering, and noise-canceling earbuds.
Photopolymerization	The microfluidic device, lab on a chip, data storage, electromagnetic interference (EMI) protection, and light-emitting diode (LED) sensing.
Continuous fiber Placement	Aircraft horizontal and vertical stabilizers are multi-purpose composite structures incorporating sensors and circuitry.
Sheet lamination	Molds, toy models, and lightweight structures for the aerospace, sports, and medical industries can all benefit from having tooling and master patterns created for them.
Polymer ink deposition	Flexible electronic devices, and supercapacitors.
Powder bed fusion	Bio-applications that carry loads, optics, supercapacitors, purified water, and fiber-reinforced wheel suspension.

**Fig. 10:**
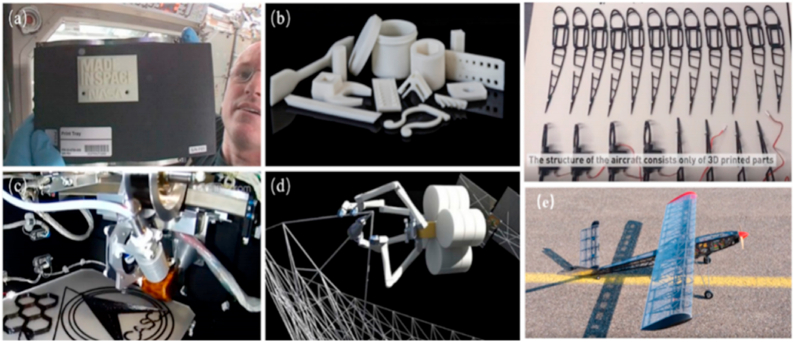
a) 3D printing in space [108], b) PLA in its purest form [109], c) PLA composites reinforced with continuous carbon fiber [108], d) Spider Fab for building massive structures [110], e) a drone construction printed in 3D using CFRCs [108].

However, CFRCs' high stiffness and specific strength have made them prominent in the aerospace industry. Making CFRCs via 3D printing was a successful approach for fabricating complex composite structures. In 2014, NASA finished the first-ever 3-D printing research in space with the assistance of Made aboard orbit, producing over 20 pure PLA samples aboard the ISS (Fig. 10(a) & b). In 2020, researchers from Xi'an Jiaotong University and China's Academy of Space Technology successfully conducted the country's first 3D printing experiment aboard a spaceship using continuous carbon fiber reinforced PLA composites (Fig. 10(c)). An enormous helical structure would be built by a space robot using continuous carbon fiber reinforced PEEK composites, according to NASA's SpiderFab idea (Fig. 10(d)). The CMASLab at ETH Zurich created and tested a morphing drone made from 3D printed CFRCs (Fig. 10(e)). displays the results of controlling roll, pitch, and yaw using only morphing control surfaces. High vacuum, massive temperature differences, and strong radiation are just a few demanding conditions that 3D-printed CFRCs must endure in the aerospace industry [108].

### 6.2 Ballistic industries

**Fig. 11:**
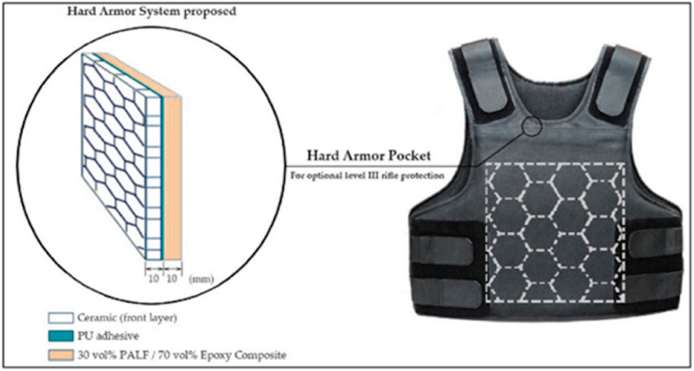
Illustration of the ballistic armored suit [111].

Gun violence poses a serious threat to society, especially to the lives of warriors and public troops. The MAS (Multilayer Armour System) is an excellent defense device. The MAS was constructed using various typical synthetic materials, including Kevlar fibers (Dyneema) and aluminum. Regarding potential applications in MAS and other branches of engineering, NFRPCs are among the most exciting new materials. These NFRPCs are part of the multiagent system's second layer. Different concentrations of figure fibers have been investigated. Results show that polyester composites with fiber volume fractions of 30% can be used as a Kevlar replacement due to their great structural integrity and energy absorption after impact [111]. Fig. 11 illustrates a diagrammatic representation of the ballistic hard armor system.

The ballistic performance of NFRPCs made from piassava fiber was analyzed. The volume fractions of the composites generated ranged from 10% to 50%. For the ballistics evaluation, 7.62 mm ammunition was used. The depth of penetration was used as a criterion to deduce whether or not a ballistic testing system satisfies the requirements of NIJ standard 0101.06. As a result of the ballistics tests, the cracked materials were disintegrating. Finally, the results of these tests demonstrated that MASs with a layer of piassava fiber composites at the top offer superior defense. This indicates the viability of using environmentally friendly piassava fiber in protective systems.

Ballistic nanocomposites have been extensively studied. Nano clay in the matrix system created glass-epoxy nanocomposites. Tensile failure in basic yarns and delamination absorbed energy. Glass-epoxy composites with graphene and nano clay were studied. These nanoparticles increased delamination, especially at the laminate's rear, but improved high-velocity impact resistance [106].

Incorporating nanoparticles improved the laminate's capacity to withstand ballistic forces and absorb more energy. The use of nanoparticles improves not only fiber-matrix adhesion but also fiber-matrix interface bonding. The impact of graphene oxide on aramid fabric was assessed by measuring its ballistic performance. Compared to uncoated fibers, the energy absorption was greatly improved because of the graphene oxide coating. Frictional increases between the fibers were blamed for this behavior. Secondary fiber deformation and microfibrillation were two of the damage processes identified. Because the fibers' strength is focused along the course of the fiber or the longitudinal direction, they would not provide much protection from a ballistic hit in the transverse direction. A coating of UHMWPE containing nanoparticles of silica was applied to them to improve the fibers' transverse mechanical properties. Coating the fibers made them harder and raised their transverse Young's Modulus. The coefficient of friction was raised after SiO_2_ nanoparticles were included. The coating did more than just boost the PPTA fibers' mechanical qualities; it also shielded them from environmental risks that could reduce their performance. An epoxy matrix dispersed with silica nanoparticles and one reinforced with carboxyl-modified multi-walled nanotubes of carbon (COOHMWCNTs) were investigated for ballistic impact performance. The composite system's COOHMWCNT count increased energy absorption. The ballistic limit velocity and energy absorbed were maximized at 1 wt% silica nanoparticle additions in composites but decreased at higher concentrations.

The effectiveness of 3D-printed armor plates made from alumina has been investigated. The ballistic performance of two plates, one made with PSD and one with DIW, was compared. When subjected to a ballistic projectile, the DIW plates outperformed the PSD plates in terms of impact resistance, hardness, and flexural strength. Small grain sizes may account for the DIW plates' toughness. For comparison, these plates' functionality was measured against that of isostatically pressed alumina plates. The pressed plates were more effective at stopping the projectile by 45 percent and had stronger flexural strength than the other plates. 3D printing introduces faults along with the part itself, which may explain why 3D-printed plates don't perform as well as pressed plates. Investigate the effectiveness of a protective shell made with additive manufacturing in withstanding impact forces.

**Fig. 12:**
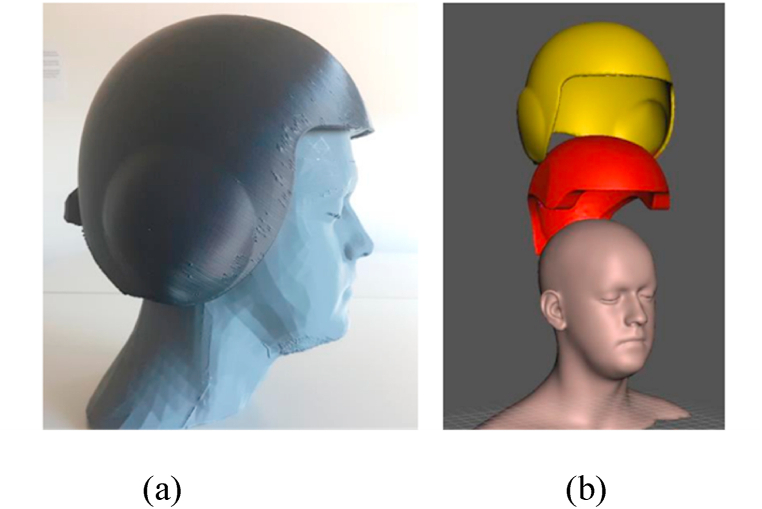
(a) 3D demonstration photo (b) The helmet features a hard shell (yellow) and insertion (red) to facilitate suiting [112] (For interpretation of the references to color in this figure legend, the reader is referred to the Web version of this article.)

In Addition, ballistic testing of various armor types compares bio-inspired and classical structures. 3D printing cannot produce a functional ballistic device. Testing reveals technology improvements. A theoretical application for armor was devised in addition to assessing the functional performance of the 3D-printed composites. Assuming future technology can address the existing limits of 3D printing, this concept highlights some of the distinct benefits of using 3D printing in the creation of personal armor. The little material waste and low active labor involved in printing items are apparent advantages. Many believe that 3D printing is better suited for customized, small-batch production than mass productionas shown in Fig. 12(a and b). The concept item highlights the ease with which (hard) body armor may be created to perfectly suit the user, demonstrating one of the characteristics of 3D printing. The design also demonstrates how simple it is to enhance a product with new (complicated) features without investing heavily in new machinery [112].

### 6.3 Textile industries

**Fig. 13:**
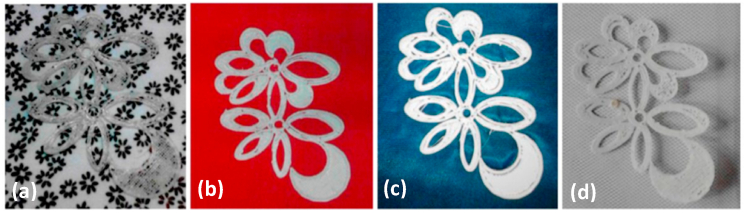
Floral 3D printing pattern, (a) cotton net, (b) wool net, (c) viscose net, and (d) polyester net [115].

To create prototypes or functional products, 3D printing is used for textiles in a variety of ways. The precision with which it can combine sophisticated design and detailing may make it preferable to more traditional printing methods in some cases. The roughness of natural fibers makes them ideal for 3D printing because of the various features they exhibit depending on their origin. It was found that the samples' hairiness, roughness, hydrophobicity, and wettability all played a role in the printing paste's success at adhering. Because natural fibers are hydrophilic, print paste penetrates fabric and sticks well. Wool fibers adhere well due to their rough surface [113]. Cellulose acetate and other biomaterials are used as printing paste on cellulosic fabrics including undyed viscose, knitted cotton, and cotton in an all-cellulosic printing process. The capacity to print on and the adhesion to the surface of most natural materials are both satisfactory [114]. Fig. 13(a–d) displays a 3D-printed flower motif on several types of fabric [115].

The capacity to print on textiles relies on their topographical characteristics. Because the print paste needs a rough surface to adhere to, it might be difficult to print on textile substrates that are too thick and smooth. Using the SLA method, 3D parts were successfully printed on a variety of knit fabrics manufactured from synthetic textile fibers such as polyester, polypropylene, polyethylene terephthalate (PET), and polyurethane. The printed polymer can more easily penetrate the knitted substrate's more porous surface structure.

Polyester from PET polymer is a popular synthetic textile. Although combined with natural substrates to create a durable, wrinkle-free, tear-resistant textile substrate, shrink-reduced, 100% woven, or knit structures are also popular. Table 15 displays 3DP on natural and synthetic textile substrates.

**Table 15:** Three-dimensional printing exemplar on both organic and synthetic textiles [114].

Fiber Source	The substrate upon which a print exists	Fabric Composition	Printing Technique
Natural	Flexible acetoxy-propyl cellulose (APC) Rigid cellulose acetate	Knitted cotton (100% cotton, single knit, 155 g/m^2^) Woven viscose ((BB12), Bamboo Plain Ivory, 140 g/m^2^,100% viscose) Woven cotton (100% cotton, plain weave, 150 g/m^2^)	Direct 3D print
Natural	ABS and Filaflex	100% Cotton, Woven	FDM
Synthetic		100% PET, Woven 100% PET, Knitted	
Natural	Transparent 3DP 405 nm UV resin	100% Cotton, Woven 100% linen, Woven	SLE
Synthetic		Weft knitted/coated (100% polyurethane) Weft knitted (100% polyester) Weft knitted/coated (100% polyurethane) Warp knitted (100% PET)	
Synthetic	Dielectric ink (a dispersion of titanium dioxide particles), Doped zinc sulfide pigment as luminous ink, Carbon-containing counter electrode ink	Woven polyester fabric	Direct 3D print

**Fig. 14:**
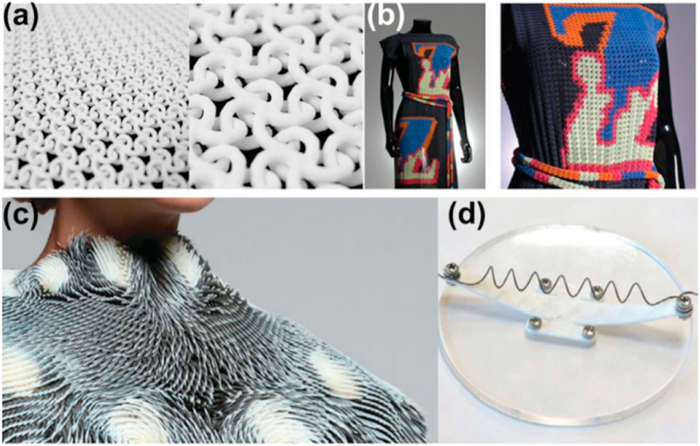
(a) depicts a chainmail garment made from interconnected Modeclix links (b). Fabric that the Caress of the Gaze can activate (c) using SMAs implanted in PLA structures created using (d) 3D printing [118].

The major objective of implementing or adopting 3DP in textile manufacturing should be to imitate essential textile qualities, including softness, flexibility, strength, and porosity [116]. Since the majority of typical textiles' yarns or fibers rest in the fabric's plane, and since the fabric's cross-plane dimension (or thickness) is a relatively small comparison to its in-plane (width and length) dimensions, ordinary textiles are categorized as 2D planar structures. The thickness of fabrics produced via 3D braiding, weaving, or knitting is significantly increased, and the threads that make up the fabric are placed systematically along the thickness axis. All braiding, weaving, and knitting in three dimensions have the same “3D″ local structure [117]. Even while these techniques have the potential to create formed objects, none of them are currently practical for making clothing. Without the limitations of traditional production processes like knitting and weaving, fashion designers and designers of functional wearables benefit greatly from the capacity to generate fully fashioned textiles. Fig. 14 illustrates printed fashion pieces made from stiff components or varying textiles. See (Fig. 14a & b) for a drape dress made from PA and SLS with Modeclix chainmail links measuring (9.5 × 9.5 × 3.6) mm. 3D printed fluidic wearables with evaluated clear and apparent regions that may generate different sounds and shades (Fig. 14(c) & d); thin woven lines produced by combining thermoplastic polyurethane (TPU) with SLS to create a lace-textured dress called Voltage-dress; incorporating shape memory alloys (SMA) in multi-material 3DP (accuracy of 16 m) to develop a flexible mesh capable of actuating and responding to a person's gaze; 3D-printed materials appear like textiles but are not [118].

### 6.4 Electronic industries

A conductive composite is any composite with a high electrical conductivity level. Conductive composites are made when insufficient quantities of polymeric resin are mixed with a filler that conducts electricity, particularly carbon-based compounds like graphene. The conductive filler particles in the composite start interacting with each other as the filler content increases, forming a continuous route that allows electrons to move freely and conduct electricity. These conductive composites can withstand corrosion, aren't heavy, and can be shaped to meet specific needs. As a result, these conductive composites can substitute metals in certain uses. Coatings, batteries, sensors, electrodes, and materials to insulate from electromagnetic interference are just a few of the many uses for conductive composites.

Manufacturers are seeing its amazing potential as 3D printing develops and becomes more prevalent in research, technology, and industry. Integrating conductors into 3D printable devices has made fundamental electronic devices like active electronic materials, electrodes, mass customization, and adaptable design possible [119]. Fused Deposition Modeling 3D printing makes the 3D electrode cheap and quick. The 3D electrode's architecture and surface area are significantly more flexible than copper, aluminum, and carbon electrodes. The 3D electrode printing technology is fully automated and precise, allowing eight electrodes to be printed in 30 min [3].

**Fig. 15:**
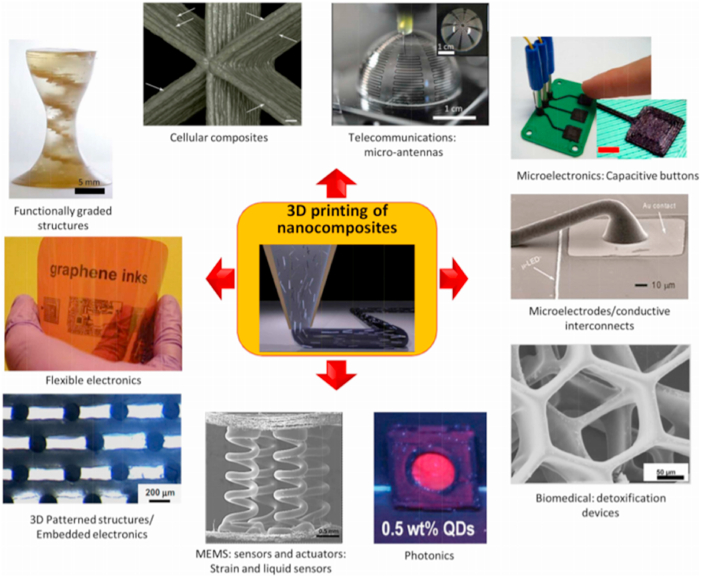
Several fields of study, including microfluidics, microelectromechanical systems (MEMS), engineered composites, microelectronics, and telecommunications, are represented by examples of 3D nanocomposite macro- and microstructures produced by various 3D printing technologies [121].

MEMS, lab-on-a-chip, engineered materials and composites, microfluidics, tissue engineering, microelectronics, and photonics have all recognized value in nanomaterials with the new printing nanocomposite materials portrayed in Fig. 15. For SC-3D printing of 2D and 3D structures for fluid sensing and EMI shielding, Serizawa et al. reported the production of highly conductive nanocomposite components (up to 5000 Sm^-1^). A ball mill blended 20 wt% polylactic acid (PLA) and carbon nanotubes. Printing proved difficult with nanotube loadings up to 40% (extrudable through the printer's tiny nozzle) [120]. Based on their findings, printed nanocomposites as 3D scaffolds significantly outperformed their hot-pressed solid counterparts in terms of their ability to block electromagnetic interference (EMI). The authors argued that in addition to antistatic coatings and flexible electrodes, there are several other uses for this class of conductive materials.

### 6.5 Biomedical

More advances in AM techniques, devices, and materials might open new possibilities for use in the medical and dental fields. SLM, SLA, FDM, and DLP are just some 3D printing processes that have found applications in the dental field. Doctors can understand a difficult structure before surgery using a 3D-printed skull or another model [122]. Powder bed fusion of metal implants, medical phantoms, biomaterials in medical additive manufacturing, and additive manufacturing of medical instruments applications involving regenerated tissue and organs are all examples of additional AM processes and areas of use [123]. The use of individualized surgical guidance is made possible by medical rapid prototyping (MRP). The research found that the MRP-assisted CSGs were the most efficient surgery method in terms of time, accuracy, and cost.

Nanotechnology and its components can replicate biological tissues' electrical conductivity, responsiveness, and chemical activity when printed in 3D. Biopolymers, including collagen and chitosan packed with osteoconductive calcium phosphates (Ca-Ps) nanoparticles, have created bone-like scaffolds. Graphene increases the electrical conductivity of polylactide-*co*-glycolide FDM scaffolds, stimulating cell development. Nanomaterials on a 3D printed framework can promote bioactivity in biomedical and lab-on-a-chip systems. Dermanaki Farahani and Dubé et al. described bone development FDM-printed scaffolds with chemically functionalized bioactive hydroxyapatite nanoparticles (nHA). The printed scaffold helps differentiate vascular cell growth and bone, while the nHA nanoparticles stimulate full cell proliferation. nHA-conjugated scaffolds enhanced human mesenchymal stem cell proliferation, adhesion, and osteogenic differentiation [121].

**Fig. 16:**
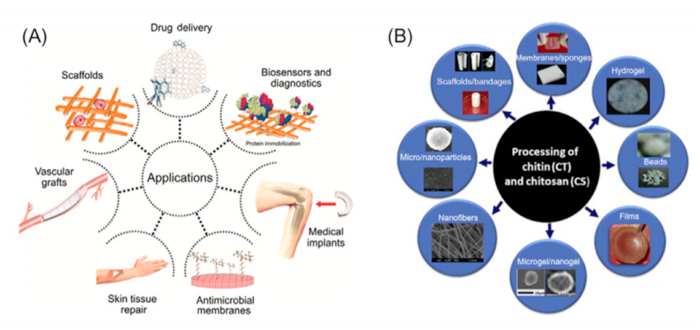
Nanocellulose in medicine (A) and a simplified example of chitin and chitosan's possible transformation into nanofibers and nanoparticles (B) [69].

In the context of a nanotechnological approach, polysaccharide-based nanocomposites have garnered a lot of interest because organic biomedical agents show good inhibition efficiency and a broad spectrum of activity but also relatively low stability (e.g., low decomposition temperature and short life expectancy). Therefore, there is a pressing need to create nanocomposite materials based on biopolymers for use in medical settings as tissue engineering scaffolds, drug delivery vehicles, wound dressings, and antimicrobial films. Researchers have been focusing on the nanofibrils, the highly crystalline portions of these natural fibers, to make cellulose and chitosan nanoscale polymeric assemblies to meet this demand, as shown in Fig. 16(a and b) [69].

In Addition, cellulose nanofibers are the most promising contender among polysaccharides for use in biomedicine. Microcrystalline cellulose (MCC), salt forms of cellulose, and modified cellulose types have long been employed in medicinal formulations (e.g., hydroxypropyl methylcellulose). However, cellulose nanofibers are an emerging technology that stands in stark contrast. Because of how its molecules are naturally arranged, cellulose nanofibers have several desirable characteristics that cannot be found in any other solid-state form of cellulose. Such features include mechanical qualities, surface chemistry, and barrier properties. There are many hydroxyl groups on cellulose's surface, making it chemically amenable to additional surface modification. Due to these features and the fact that cellulose materials are biocompatible, they show considerable potential in numerous biotechnological and biomedical settings.

**Fig. 17:**
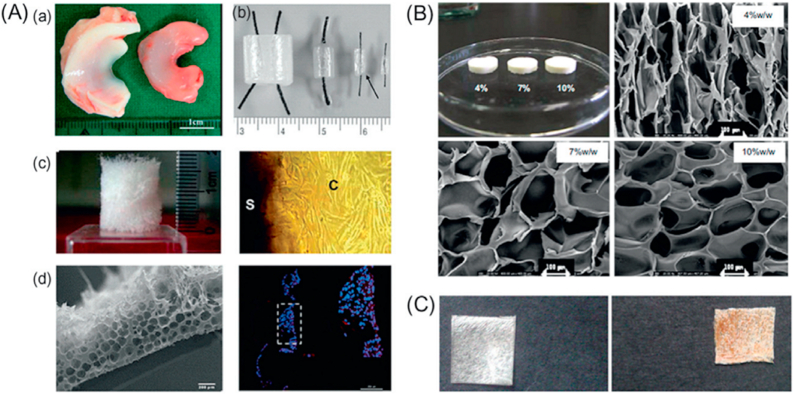
Bioengineering materials (A) based on boron nitride (BNC). BNC meniscus implant, (a). b) BASYC tubes of varying diameters. (c) Phase-contrast micrograph of fibrous cells growing on a BNC sponge used in tissue engineering after five days of in vitro culture (S, scaffold; C, cells). (d) A scanning electron micrograph (SEM) of a BNC scaffold seeded with human urine-derived stem cells and labelled with -smooth muscle actin (scanning electron microscope; scale bar: 200 m); (B) a photograph and scanning electron micrographs (SEM) of sponges prepared with chitosan solution at different concentrations on the vertical side (magnification of 200), and (C) a photograph of a chitosan scaffold and a TGF-3-loaded chitosan scaffold [69].

Various BC/collagen biocomposites and BC/heparin biocomposites have been produced for tissue engineering. Biofill, Bioprocess, and X Cell are just a few of the modern companies that sell BC for use in wound care shown in Fig. 17(A-C).

In addition, researchers at Romania's Polytechnic University of Bucharest developed and tested a three-dimensional, multilayered graphene biosensor to aid in the development of more accurate and reliable biosensors for use in individualized healthcare and treatment solutions. Although graphene with multiple layers is far less reactive than single-layer graphene, the material's edge is far more reactive than its surface. Because graphene is relatively innocuous, biosensors made of multilayered graphene are a great choice. When designing a biosensor, it's crucial to think about both the biosensor and the interface it will use. The human skin serves as the interface here. Physical and chemical data can be collected from human skin, which has the biggest surface area of any organ in the body and responds differently to internal and external stimuli. Amphiphilic pyrrole monomer-glucose oxidase combinations adsorbed on platinum electrodes were oxidatively polymerized to produce highly sensitive glucose biosensors. Endogenous (ascorbate and urate) and exogenous (paracetamol) substances that are electro-oxidizable caused significant interferences with these sensors, which were based on H_2_O_2_ electro-oxidation at 0.5 V vs shape-changing effect (SCE). The goal of fabricating these bilayer structures was to reduce interferences by integrating the outer layer of the previous polymer film with the inner layer of electrogenerated poly (phenylene diamine), Nafion. The Nafion-based biosensor demonstrated a glucose sensitivity of 0.4 mA/M/cm2, with urate, ascorbate, and paracetamol causing almost no interference.

### 6.6 Sporting

The versatility of Carbon fiber reinforced plastics (CFRP) is a huge plus for the sporting goods industry. It's commonly used to craft ski boards, rackets, fishing rods, golf clubs, bows and arrows, bicycles, baseball bats, boats, ice hockey sticks, and rowing oars. The CFRP and hierarchical composite must evaluate the structure of each sporting good according to its own set of criteria. When used in golf balls, for instance, composites extend the balls' longevity by enhancing their damping function and decreasing their dead weight. The racket's composite structure allows for a tighter string with greater specific strength and modulus, allowing faster serves and reduced shock during rebuffs. Carbon fiber reinforced plastics (CFRPs) guarantee perfect shock absorption and top speed with minimum effort for bicycles. Additionally, “NAWAStich” is a new development in the wheel of a terrain racing bicycle that combines CNT with CFRP to improve the wheel's resistance to impact damage and its strength relative to regular CFRP. This occurred because of the increased buckling resistance inherent in the rim's interior during the high compression phenomena [124].

However, most major automakers worldwide have studied NFPCs extensively to adopt them into their products. European automakers have researched various automotive interior applications, including boot linens, seat backs, front and rear door linens, parcel shelves, door-trim panels, and truck linens. Natural fiber incorporated in polymers is in high demand for applications beyond vehicle interiors, such as the space between the headlights and the fender of a passenger bus.

### 6.7 Robotics

**Fig. 18:**
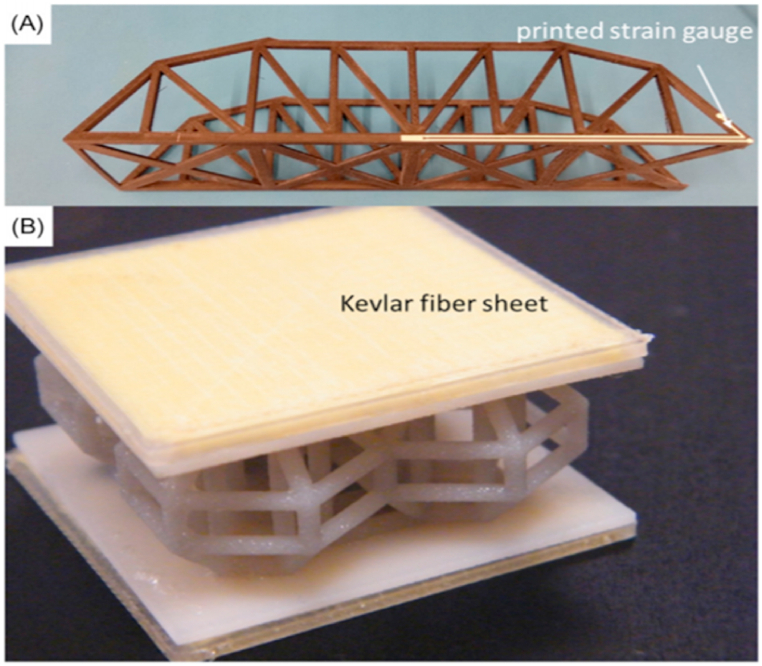
(A) A model of a truss bridge made of carbon fiber, complete with 3D-printed strain gauges, and (B) a sheet of Kevlar fiber sandwiched between two layers of nylon [126].

To get beyond the limitation of flat-surfaced production, a device capable of multi-axis 3D printing is needed to manufacture complex products with curved or cylindrical geometries. As a result of this study, a novel composite production process has been developed that combines multi-axis 3D printing with composite materials to produce three-dimensional things [125]. To eliminate the need for the laborious and expensive hand-laying procedure in composite manufacturing, automated fiber placement (AFP) and automated tape laying (ATL) have been developed and are currently in testing. Thus, it is significant that these drawbacks can be avoided using the robotic 3-D printing procedure for composites. Multi-axis 3D printing with thermoplastics has been the subject of numerous investigations. However, current research on composite manufacturing focuses on utilizing short fiber additions [122]. Using 3D printing, scientists have studied the mechanical properties of items reinforced with short fiber additions, printed using a 5-axis machine with rotating heads. However, a two-axis turntable is needed for this technique to maintain a steady gravity-deposition direction. This method is quite costly and complex to implement for control [126]. In contrast, conformal printing with robotic arms can print electronics and sensors directly onto composite materials, resulting in multifunctional composite parts shown in Fig. 18(A,B).

**Fig. 19:**
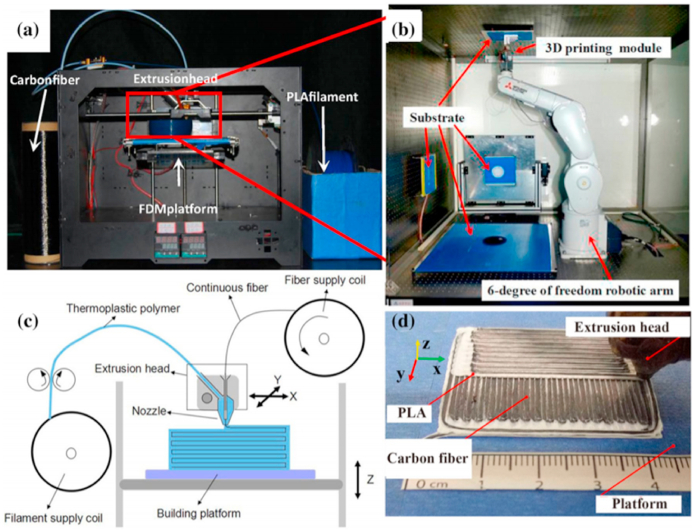
Images (a) of an FDM 3D printer, (b) of a 3D printing robot, (c) of a flowchart depicting the 3D printing process, and (d) of produced specimens [127].

While CF-reinforced composites made with traditional CF lamination techniques are effective, 3D-printed CF composites offer several benefits. For instance, 3D printing cuts down on time and money because it eliminates the need for mold and uses gentle process conditions. In contrast, hot-press molding, which makes use of molds and substantial pressure was used to create the composites in earlier works. Second, 3D printing makes it simple to create complex shapes like hollow or conformal ones. Third, using a 3D printing robot and 3D printing processes, integrated manufacture of big components is possible, negating the need for assembly Fig. 19(a–d). Advantages in processing ensure that composites remain cost-competitive with alternative EMIS materials. In addition, several other types of matrix materials can be used for 3D printing. Matrix materials can be made from various thermoplastics, including PLA, ABS, PC, PP, PS, PA, and PEEK. Multipurpose shielding materials can be easily prepared.

Polyjet AM with multi-material fabrication enhances mechanical properties including stiffness, heat resistance, strength, and durability, making it promising. Filler materials for 3D-printed multi-material structures include particles, continuous and discontinuous fibers. These manufactured structures have been intensively investigated as an alternative to complex and expensive composite constructions. Multi-material polyjet 3D printing has advanced. Recent developments include multi-material polyjet printers that can make a variety of prototypes and industrial items.

Stratasys J 735TM, Objet 1000 plusTM and Stratasys 750TM are popular production series polyjet 3D printers. They offer the most agility, beauty, size, and artistry at every production stage. Multi-material AM employing polyjet technology improves dimensional conformance between suggested designs and produced structures at various scales. Bio-inspired polymer composites, soft robotics, and four-dimensional printing have enlarged design space and solved manufacturing limits. Integrating soft and hard materials into some single end-use products improved mechanical properties like flexibility and hardness in the additively produced composite framework inspired by natural composites.

## 7 Environmental Impact of Fiber reinforced composite

Researchers and structural designers have made significant strides in recent years to enhance the polymer composites' resistance to the elements and their ability to withstand damage. These materials are subjected to arduous environmental conditions throughout their service lives, compromising their durability and integrity. Environmental exposures can cause minute changes at the fiber/polymer interface, and these changes might eventually expose the weakest zone and threaten the composite's structural integrity. Timely verification of a variety of micro-characterization methods and accurate modeling are required to forecast the materials' durability in long-term applications [128]. However, due to rising environmental and sustainability concerns, natural fibers (NFs) are being used more frequently because of their excellent sustainability, biodegradability, eco-friendliness, and cost-effectiveness. Outstanding mechanical capabilities equivalent to glass-fiber sheet molding compound (GFSMC) have been demonstrated by NFRCs. Utilizing kenaf fiber (Hibiscus cannabinus, L. Malvaceae) as a renewable NF resource is a top priority because of the abundant kenaf supplies that could be used to manufacture high-quality goods. The remarkable recent development of kenaf fiber composites can be attributed to the material's eco-friendliness and great economic qualities. High water uptake of NFs and poor mechanical performance of NFRCs have been major concerns due to dangling polar groups (e.g. hydroxyl) in NFs, which weaken the bonding strength of the interface between the fibers and the polymer [84].

Natural fibers are becoming a viable alternative to synthetic fibers in polymer composite systems. Environmental issues like climate change have prompted engineers to incorporate renewable materials in composite buildings. Natural fibers' hemicelluloses, cellulose, and lignin faults reduce their compatibility with polymer matrices. Chemically modified fiber surfaces may improve fiber-matrix compatibility. As the industry seeks to minimize its dependence on petroleum-based fuels and goods, it must study other environmentally friendly, sustainable materials. The sustainability (energy economy, emission) of NFRPC component production has also been briefly explored. In the automotive, transportation, construction, and packaging industries, NFRPCs have been confirmed as a substitute for SFRPCs [129]. Since natural fiber manufacturing is labor-intensive, the NFRPC industry will create new jobs and help emerging countries reduce poverty. Global warming concerns have shifted attention to sustainable natural fiber composites.

In numerous industrial settings, fiber-reinforced polymers have actively replaced more traditional materials. But after their useful life is over, the dilemma of what to do with these materials emerges. Recycling polymers isn't exactly ground-breaking right now, but many people are trying to figure out how to make composites and nanocomposites more recyclable, which might have huge implications. New materials called hybrid composites combine two or more reinforcing particles or fibers [130]. Adding NanoSiO_2_ particles to wood-plastic composites improves their qualities and makes them more profitable for large-scale applications, although these materials already have many potential uses. Because of their reduced solidity, lower cost, and low density, natural fiber-reinforced polymer composites are an attractive alternative to synthetic composite goods for commercial applications including buildings, the automobile sector, and constructions. Designs of automotive gears frequently use natural fiber as a foundational material rather than metal because of the high energy consumption involved in creating metal components, which affects the environment.

Natural fiber composites may have fewer negative effects on the environment than glass- or carbon-fiber composites if their production impacts are minimal enough, or if the product's environmental performance is improved [131]. Life-cycle assessment (LCA) considers emissions into the environment, resource consumption, and land use to conclude environmental impacts like stratospheric ozone depletion, climate change, eutrophication, tropospheric ozone, acidification, and toxicological stress on human health and ecosystems. The environmental benefits of composite materials over conventional aluminum airplane structures have been proved and studied in life cycle assessment (LCA) research [111].

Environmental life cycle assessment (LCA) comparison of plastic pallets made from various ReCiPe-based bio-composites and composites. Eco-efficiency was also quantified using the environmental assessment results, which corroborated a link between the various types of environmental impact and the outcomes [132]. Using LCA, we used two distinct cellulose extraction methods to compare the environmental effects of thermal insulation pads made from rice straw. The thermal steam explosion method was found to have fewer negative effects on the environment than the chemical extraction method since it lowered eco-toxicity and increased fiber production. The thermal explosion procedure had the disadvantage of requiring more power than alternative methods [133].

The life cycle assessment showed that compared to petroleum-based products, bio-based polymers' environmental consequences and energy consumption were significantly better [134]. We looked into the effects the kenaf board has over its whole lifespan by contrasting its functionality with that of other, more easily replaced materials including polyurethane foam, flax rolls, glass wool, mineral wool, stone wool, and paper wool. Synthetic materials were shown to have the greatest impacts, while mineral wools were responsible for the best performances [135]. LCA analysis revealed numerous social, environmental, and economic benefits from using natural jute fibers in place of glass fibers to manufacture the structural frontal bonnet of an off-road vehicle [17].

**Fig. 20:**
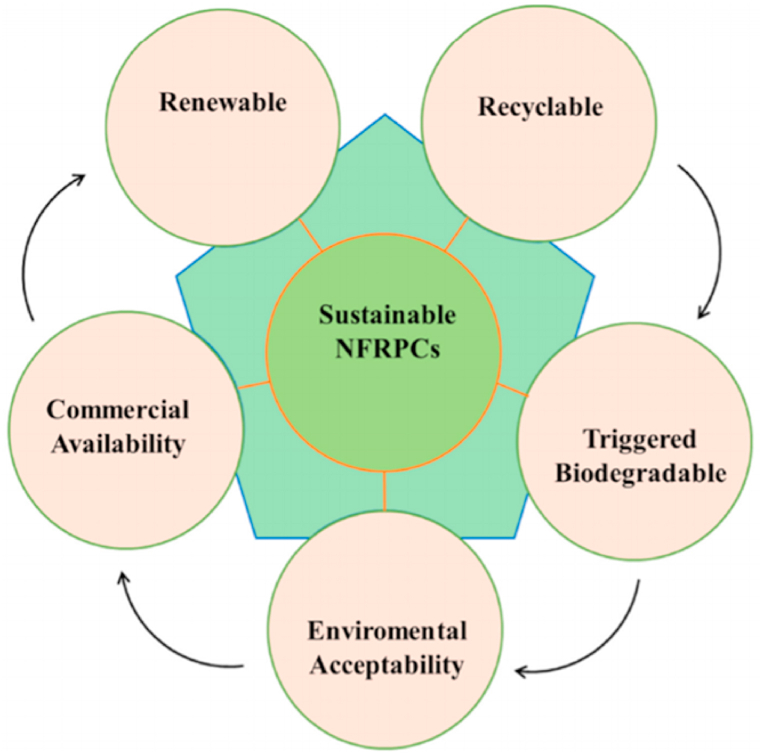
Characteristics typically linked with NFRPCs [111].

When NF composites are burned, they provide carbon credits and reduce the environmental risk. The NFs and LCA residues that contribute to global energy requirements (GER) are depicted in Fig. 20.

## 8 Conclusion and prospects

Compared to more conventional production methods, additive manufacturing for FRP composites has many advantages. It allows for the creation complex geometries and shapes with improved accuracy and precision. The ability to customize parts and products based on specific requirements also makes it a valuable tool for various industries. Moreover, additive manufacturing of FRP composites reduces material waste and enables the use of sustainable materials. It also improves the mechanical properties of the final product, such as its stiffness, strength, and durability, and improves the performance of the composite material. However, several challenges must be addressed before it can be widely adopted. These challenges include material selection, fiber alignment, print defects, post-processing, and quality control. The cost of AM equipment and materials can also be a limiting factor for small-scale production. Despite the challenges, AM for FRP composites has the potential to revolutionize the design and manufacturing of lightweight and high-performance structures. Further research and development in this field can produce cost-effective and sustainable FRP composite products for various industries, such as automotive, aerospace, and construction. Here are some possible avenues for future research in this field.

I. Material Development: Combination of different types of fibers (e.g., glass, carbon, aramid) with different types of polymers (e.g., epoxy, nylon, polypropylene) to achieve the desired thermal, mechanical, and electrical properties. Additionally, researchers can explore the use of nanomaterials, such as graphene, to improve the performance of FRP materials.
II. To further optimize the manufacturing process, we can examine how changing factors in the manufacturing process (such as temperature, printing speed, and layer thickness) affect the mechanical properties of FRP composites.
III. Design for Additive Manufacturing: We can investigate new design strategies, such as topology optimization, to create optimized parts for AM. Investigate the potential of multi-material printing, which enables combining materials and characteristics in a single component.

Combining composite materials helps diversify and enlarge filament feedstock for stronger mechanical properties. A continuous fiber processing approach allows the fabrication of natural fiber-reinforced polymer composites as an alternative to glass and carbon fiber reinforcements. Other research can improve polymer-fiber interfacial interaction through chemical and physical treatments. For a sustainable future, an environmentally friendly filament feedstock like recycled plastic blended with filler or bio-waste fiber might be tested. This effort gives a database that may aid industrial growth and 3D printing composites.

## Author contributions

Noshin Tasnim Tuli: Writing – original draft, Visualization, Conceptualization. Sinthea Khatun: Writing – original draft, Visualization, Methodology. Adib Bin Rashid: Writing – review & editing, Visualization, Supervision, Conceptualization

## Data availability statement

Data will be provided on request.

## Declaration of competing interest

The authors declare that they have no known competing financial interests or personal relationships that could have appeared to influence the work reported in this paper.
